# Protein phosphorylation differs significantly among ontogenetic phases in *Malus* seedlings

**DOI:** 10.1186/1477-5956-12-31

**Published:** 2014-05-25

**Authors:** Yan Wang, Yi Wang, Yong Bo Zhao, Dong Mei Chen, Zhen Hai Han, Xin Zhong Zhang

**Affiliations:** 1 Institute for Horticultural Plants, China Agricultural University, Beijing 100193, China; 2 Changli Institute of Pomology, Hebei Academy of Agricultural and Forestry Sciences, Changli 066600, China

**Keywords:** Apple, Vegetative phase change, Floral transition, Phosphorylated proteomics

## Abstract

**Background:**

Although protein phosphorylation is an important post-translational modification affecting protein function and metabolism, dynamic changes in this process during ontogenesis remain unexplored in woody angiosperms.

**Methods:**

Phosphorylated proteins from leaves of three apple seedlings at juvenile, adult vegetative and reproductive stages were extracted and subjected to alkaline phosphatase pre-treatment. After separating the proteins by two-dimensional gel electrophoresis and phosphoprotein-specific Pro-Q Diamond staining, differentially expressed phosphoproteins were identified by MALDI-TOF-TOF mass spectrometry.

**Results:**

A total of 107 phosphorylated protein spots on nine gels (three ontogenetic phases × three seedlings) were identified by MALDI-TOF-TOF mass spectrometry. The 55 spots of ribulose-1, 5-bisphosphate carboxylase/oxygenase (Rubisco) large-chain fragments varied significantly in protein abundance and degree of phosphorylation among ontogenetic phases. Abundances of the 27 spots corresponding to Rubisco activase declined between juvenile and reproductive phases. More extensively, phosphorylated β-tubulin chain spots with lower isoelectric points were most abundant during juvenile and adult vegetative phases.

**Conclusions:**

Protein phosphorylation varied significantly during vegetative phase change and floral transition in apple seedlings. Most of the observed changes were consistent among seedlings and between hybrid populations.

## Background

Post-translational modification (PTM) is a common regulatory mechanism in organisms. Protein phosphorylation, one of the most important PTMs, influences many different protein characteristics, including enzymatic activity, turnover, subcellular localization, and protein–protein interactions [1]. Protein phosphorylation is required for the proper functioning of many proteins involved in cellular processes ranging from signal transduction, cell differentiation, and development, to cell cycle control and metabolism [2]. Cell signaling mechanisms often transmit information via post-translational protein modifications, the most important of which is reversible protein phosphorylation regulated by the concerted action of protein kinases and protein phosphatases [3]. In brassinosteroids, for instance, tyrosine phosphorylation of four C-terminal residues of BRI1 is important for its kinase activity, whereas phosphorylation of Tyr831 in its juxtamembrane domain inhibits growth and delays flowering [4]. In one study, tyrosine phosphorylation levels of 19 proteins changed after abscisic acid (ABA) treatment, demonstrating the regulatory role of tyrosine phosphorylation in ABA signal transduction [5].

Phosphorylation of proteins differs significantly among tissues, organs and physiological ages. Ratios in protein complexes of the oxidative phosphorylation system have been found to differ markedly among leaves, stems, flowers, roots and seeds of *Arabidopsis thaliana*[6]. Three glutamine synthetase isoenzymes in *Medicago truncatula* can be phosphorylated by soluble protein kinases in nodules, leaves and roots, but the kinetic activity of kinases against glutamine synthetase isoenzymes from the different organs varies according to isoenzyme [7]. Some potential regulators, such as the 14-3-3 protein in rice phloem and xylem sap, bind proteins in a phosphorylation-dependent manner [8]. Phosphatase content has been found to increase along with increasing tree age in *Acacia tortilis* subsp. *raddiana*[9]. In one experiment, root surface phosphatase activity of non-mycorrhizal 3-month-old Norway spruce (*Picea abies*) seedlings was much higher than that of 9-month-old seedlings [10].

Protein phosphorylation levels may also change along with physiological status. In a study of rice cell suspensions, two 26-kDa and 40-kDa tyrosine-phosphorylated proteins were only detected during late stages of cell senescence [11]. Phosphorylation patterns change distinctly and specifically in apple fruit during senescence, with a 60-kDa membrane protein found to be phosphorylated specifically in Ca++ - treated but not in control-fruit protein fractions [12].

Two ontogenetic transitions occur in perennial woody angiosperms after seed germination: a vegetative phase change, from the juvenile phase to the adult vegetative phase, and a floral transition from the adult vegetative phase to the reproductive phase [13]. Protein phosphorylation most likely differs among these plant ontogenetic phases. In *Sequoia sempervirens*, a 32-kDa phosphorylated protein has been revealed to be adult-phase specific, while a 31-kDa phosphoprotein is specific to the juvenile phase [14]. Based on western blotting with a phosphotyrosine-specific antibody, three 25-kDa, 39-kDa and 54-kDa phosphorylated proteins were found to be more abundant in juvenile than in adult *S. sempervirens* tissues, whereas two tyrosine phosphorylated proteins of 25 kDa and 34 kDa were more highly expressed in adult than in juvenile or rejuvenated tissues [15]. PTM types and levels of some enzymes, i.e., transketolase, chloroplast protease, and heat shock proteins, are postulated to vary from one developmental phase to another [16]. Nevertheless, insufficient data are available with respect to the degree and extent of variation of protein phosphorylation during ontogenesis in woody perennials.

In this study, we investigated differences in protein phosphorylation among ontogenetic phases of apple (*Malus domestica*) seedlings. After pre-treatment of extracted proteins with alkaline phosphatase, we used two-dimensional gel electrophoresis (2-DE) followed by phosphoprotein specific Pro-Q Diamond staining to detect phosphoproteins from the different ontogenetic phases. Differentially expressed phosphoproteins were then identified using matrix-assisted laser desorption/ionization time-of-flight/time-of-flight (MALDI-TOF-TOF) high-resolution tandem mass spectrometry. Phosphorylation patterns of ribulose-1, 5-bisphosphate carboxylase/oxygenase (Rubisco) large-chain fragments, Rubisco activase, and β-tubulin were found to be clearly different among ontogenetic phases.

## Results

### Identification of phosphoproteins

**Figure 1:**
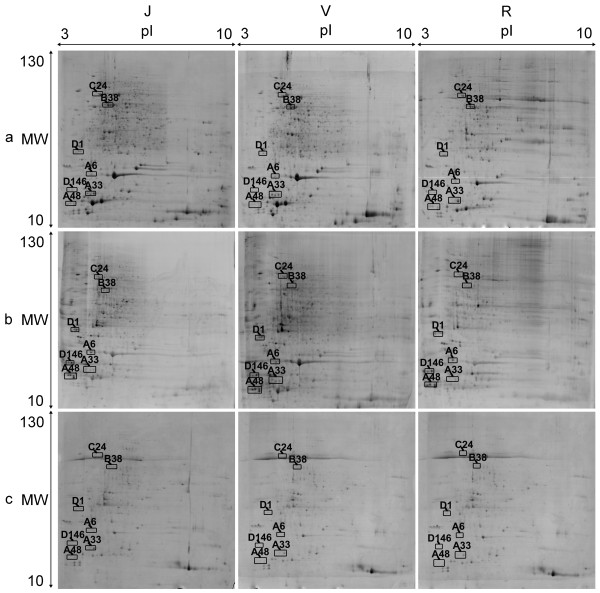
**2-DE images of differentially expressed phosphorylated proteins in different ontogenetic phases of apple seedling 02-18-081.** The seedling 02-18-081 was derived from a cross hybrid ‘Jonathan’ × ‘Golden Delicious’, the proteins were extracted from leaf sample. Juvenile, adult vegetative and reproductive phases are designated by J, V and R, respectively. Proteins were profiled in the first dimension by isoelectrofocusing using linear IPG stripes (pH 3–10, 24 cm) and on SDS-polyacrylamide gels in the second dimension. Phosphorylated protein groups are indicated by open squares. Panel a corresponds to global proteins stained with Coomassie Brilliant Blue R-350, panel b represents total phosphorylated proteins stained with Pro-Q Diamond, and panel c shows dephosphorylated proteins pre-treated with calf intestinal phosphatase and stained with Coomassie Brilliant Blue R-350. Approximate molecular masses and isoelectric points are indicated on the right edge and top margin, respectively.

We compared protein spots on nine gel images obtained from three technical replicates of the three ontogenetic phases of each seedling. Spots that matched exactly on at least eight gels and whose corresponding proteins were differentially expressed among ontogenetic phases were then selected. Of these differentially expressed protein spots, only those showing good robustness among the three seedlings were subjected to further analysis (Figure 1, Additional file 1: Figure S1 and Additional file 2: Figure S2). The selected protein spots were then compared with gel images of global, dephosphorylated and phosphorylated proteins. Out of the selected spots, seven groups of phosphorylated proteins were found to be differentially expressed among the three ontogenetic phases (Table 1, Additional file 3: Table S1 and Additional file 4: Table S2).

**Table 1:** Expression abundance of matched spots from different ontogenetic phases of three apple seedlings

**02-18-081**	**A6-1**	**A33-1**	**A33-2**	**A33-3**	**A48-1**	**A48-2**	**A48-3**	**B38-1**	**B38-2**	**B38-3**	**C24-1**	**C24-2**	**C24-3**	**D1-1**	**D1-2**	**D1-3**	**D1-4**	**D146-1**
J	0.061a	0.045b	0.045b	0.112b	0.030a	0.020b	0.025a	0.053a	0.381a	0.186a	0.010a	0.041a	0.095a	0.012a	0.006a	0.021a	0.028a	0.015a
V	0.063a	0.079a	0.057a	0.133a	0.022b	0.027a	0.018b	0.046b	0.302b	0.131b	0.008b	0.020b	0.039c	0.009ab	0.000	0.021a	0.018b	0.014a
R	0.040b	0.000	0.000	0.113b	0.017c	0.020ab	0.022ab	0.035c	0.157b	0.082b	0.007b	0.017b	0.073b	0.005b	0.000	0.028a	0.009c	0.016a
02-17-115	A6-1	A33-1	A33-2	A33-3	A48-1	A48-2	A48-3	B38-1	B38-2	B38-3	C24-1	C24-2	C24-3	D1-1	D1-2	D1-3	D1-4	D146-1
J	0.054a	0.000	0.000	0.098b	0.041a	0.040c	0.036a	0.063a	0.246a	0.165a	0.010a	0.039a	0.060a	0.000	0.000	0.046a	0.041a	0.066a
V	0.046b	0.000	0.044a	0.126a	0.015a	0.082a	0.028b	0.055b	0.196b	0.125b	0.015a	0.034a	0.094a	0.012a	0.014a	0.054a	0.027b	0.044b
R	0.037c	0.000	0.000	0.085b	0.000	0.066b	0.034a	0.046c	0.182b	0.138b	0.013a	0.027b	0.079a	0.000	0.000	0.031b	0.032b	0.025c
07-07-133	A6-1	A33-1	A33-2	A33-3	A48-1	A48-2	A48-3	B38-1	B38-2	B38-3	C24-1	C24-2	C24-3	D1-1	D1-2	D1-3	D1-4	D146-1
J	0.153a	0.182b	0.138ab	0.242b	0.139a	0.052b	0.098ab	0.175a	0.903a	0.661a	0.026a	0.030a	0.075a	0.018a	0.025a	0.083a	0.047b	0.103a
V	0.104b	0.260a	0.174a	0.341a	0.143a	0.080a	0.085b	0.128b	0.766b	0.560b	0.017b	0.026a	0.050ab	0.018ab	0.024a	0.100a	0.061a	0.083b
R	0.070c	0.211ab	0.145b	0.231b	0.091a	0.054b	0.118a	0.115b	0.693c	0.484c	0.000	0.000	0.042b	0.014b	0.018b	0.056b	0.035c	0.061c

J, juvenile phase; V, adult vegetative phase; R, reproductive phase. Data were from 2-DE gels containing global proteins. Statistical analysis using Student’s *t*-test at the *p* < 0.05 level was used to assess the significance of expression abundance differences among mutually matched gel spots from J, V and R phases within the same seedling.

Among the 146 spots from the seven differentially expressed groups, 107 spots from five groups were successfully identified by MALDI-TOF-TOF analysis (Additional file 5: Table S3; Additional file 6: Figure S3). Of the 107 validly identified spots, the 55 spots constituting groups A6, A33 and A48 were identified as Rubisco large-chain fragments. The 27 spots of group B38 were identified as Rubsico activase, while the 25 spots of group C24 were determined to be β-tubulin. Because the confidence interval (CI) values of protein spots in groups D1 and D146 were lower than 95% (Additional file 7: Table S4), these groups were excluded from further analysis.

#### Rubisco large-chain fragments

Although the spots in groups A6, A33, and A48 were identified as Rubisco large-chain fragments, expression patterns of these proteins differed considerably from one another.

**Figure 2:**
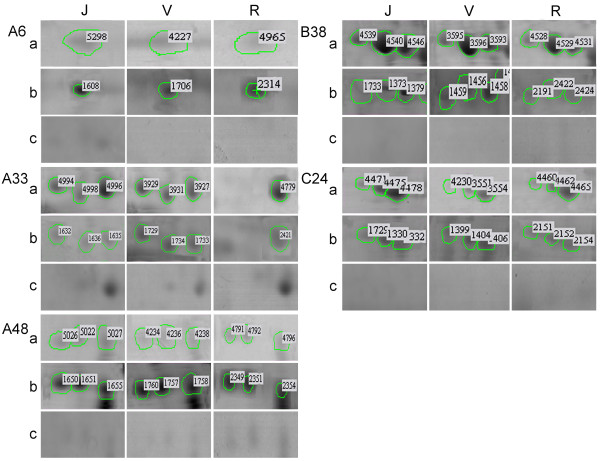
**Zoomed-in images of phosphorylated protein in different ontogenetic phases of apple seedling 02-18-081.** The seedling 02-18-081 was derived from a cross hybrid ‘Jonathan’ × ‘Golden Delicious’, the proteins were extracted from leaf sample. Juvenile, adult vegetative and reproductive phases are designated by J, V and R, respectively. Panel a corresponds to global proteins stained with Coomassie Brilliant Blue R-350, panel b represents total phosphorylated proteins stained with Pro-Q Diamond, and panel c shows dephosphorylated proteins pre-treated with calf intestinal phosphatase and stained with Coomassie Brilliant Blue R-350. In this figure, spot numbers referenced in the text and in Table 1 (with their corresponding spots in parentheses) are A6-1 (spots 5298/4227/4965 in panel a; 1608/1706/2314 in panel b), A33-1 (4994/3929/- in panel a; 1632/1729/- in panel b), A33-2 (4998/3931/- in panel a; 1636/1734/- in panel b), A33-3 (4996/3927/4779 in panel a; 1635/1733/2421 in panel b), A48-1 (5026/4234/4791 in panel a; 1650/1760/2349 in panel b), A48-2 (5022/4236/4792 in panel a; 1651/1757/2351 in panel b), A48-3 (5027/4238/4796 in panel a; 1655/1758/2354 in panel b), B38-1 (4539/3595/4528 in panel a; 1733/1459/2191 in panel b), B38-2 (4540/3596/4529 in panel a; 1373/1456/2422 in panel b), B38-3 (4546/3593/4531 in panel a; 1379/1458/2424 in panel b), C24-1 (4471/4230/4460 in panel a; 1729/1399/2151 in panel b), C24-2 (4475/3551/4462 in panel a; 1330/1404/2152 in panel b), C24-3 (4478/3554/4465 in panel a; 1332/1406/2154 in panel b).

Group A6 consisted of only one spot, A6-1 (Table 1), which was associated with a molecular mass of 19 kDa and an isoelectric point of 4.238 (Figure 1, Additional file 1: Figure S1, and Additional file 2: Figure S2). This spot was considered to be a phosphorylated protein because it was distinctly visible in Pro-Q Diamond stained gel images, but disappeared completely after treatment with calf intestinal phosphatase (Figure 2, Additional file 8: Figure S4 and Additional file 9: Figure S5: A6, panels b and c). The global abundance of this protein in the reproductive phase (spots 4965/811/312) of the three seedlings was significantly lower than in juvenile (spots 5298/679/420) and adult vegetative (spots 4227/1085/302) phases (Table 1; Figure 2, Additional file 8: Figure S4 and Additional file 9: Figure S5: A6, panel a).

Three spots were components of group A33: A33-1, A33-2 and A33-3 (Table 1). Their associated molecular masses were 16 kDa, and their respective isoelectric points on the gels were 4.092, 4.222 and 4.355 (Figure 1, Additional file 1: Figure S1 and Additional file 2: Figure S2). A33-1 expression was relatively weak during the juvenile phase of the three seedlings (spots 4994/-/345). The expression abundance of A33-2 was higher during the adult vegetative phase (spots 3931/1088/247) than in juvenile (spots 4998/-/348) and adult reproductive (spots -/-/183) phases, although the difference between adult vegetative and reproductive phases was not significant in seedling 07-07-133. The protein in spot A33-3 was expressed more abundantly during the adult vegetative phase (spots 3927/887/244) than in juvenile (spots 4996/542/346) and adult reproductive (spots 4776/694/180) phases. The expression abundance of A33-3 was greater than that of A33-1 and A33-2 (Table 1; Figure 2, Additional file 8: Figure S4 and Additional file 9: Figure S5: A33, panel a). Almost all spots were obviously stained by Pro-Q Diamond dyes. After dephosphorylation with calf intestinal phosphatase, spot A33-1 was no longer present on the gels, and spots A33-2 and A33-3 were invisible without magnification (Figure 2, Additional file 8: Figure S4 and Additional file 9: Figure S5: A33, panels b and c).

Three spots were detected in group A48: A48-1, A48-2 and A48-3 (Table 1). The related molecular mass of this group was 14 kDa, and the isoelectric points were 3.289, 3.398 and 3.546, respectively (Figure 1, Additional file 1: Figure S1 and Additional file 2: Figure S2). In all three seedlings, the expression of protein in A48-1 was relatively lower in the reproductive phase, but this difference was not statistically significant in seedling 07-07-133. A48-2 was expressed more abundantly in the adult vegetative phase (spots 4236/1095/294) than in juvenile (spots 5022/696/433) and adult reproductive (spots 4792/814/326) phases. The expression abundance of A48-3 was likewise lower in the adult vegetative phase (spots 4238/1097/295) than in juvenile (spots 5027/697/435) and reproductive (spots 4796/721/327) phases (Table 1; Figure 2, Additional file 8: Figure S4 and Additional file 9: Figure S5: A48, panel a). Almost all spots were apparently stained by Pro-Q Diamond dyes. After treatment with calf intestinal phosphatase, isoelectric points of all spots were slightly increased (Figure 2, Additional file 8: Figure S4 and Additional file 9: Figure S5: A48, panels b and c).

#### Rubisco activase

The three spots in group B38 were identified as fragments of Rubisco activase. The related molecular mass of this group was 47 kDa, and the associated isoelectric points were 4.762, 4.894 and 4.999 (Figure 1, Additional file 1: Figure S1 and Additional file 2: Figure S2). The expression of spot B38-2 was more abundant than that of B38-1 and B38-3 during all ontogenetic phases of the three seedlings. Expressions of all three spots during the juvenile phase were significantly higher than their corresponding partners in adult vegetative and reproductive phases (Table 1; Figure 2, Additional file 8: Figure S4 and Additional file 9: Figure S5: B48, panel a). All spots were unambiguously present on the Pro-Q Diamond stained gels, but completely disappeared after treatment with calf intestinal phosphatase (Table 1; Figure 2, Additional file 8: Figure S4 and Additional file 9: Figure S5: B38, panels b and c).

#### β-Tubulin chain

Protein spots in group C24 were matched to β-tubulin. The related molecular mass of this group was 56 kDa, with isoelectric points of 4.444, 4.511, and 4.574 (Figure 1, Additional file 1: Figure S1 and Additional file 2: Figure S2). Protein in spot C24-3, with a higher isoelectric point, was expressed more abundantly than spots with lower isoelectric points during all three phases in each seedling. The expressions of proteins in all three spots were significantly lower during the reproductive phase than in the juvenile phase and the adult vegetative phase, except that no significant difference was detected in the abundances of spots C24-1 and C24-3 between ontogenetic phases in seedling 02-17-115 (Table 1; Figure 2, Additional file 8: Figure S4 and Additional file 9: Figure S5: C24, panel a). Almost all spots on the gels were stained by Pro-Q Diamond, but disappeared after treatment with calf intestinal phosphatase (Figure 2, Additional file 8: Figure S4 and Additional file 9; Figure S5: C24, panels b and c).

## Discussion

Protein phosphorylation varied significantly during ontogenetic development in apple seedlings. First, phosphorylation levels of individual proteins changed markedly during ontogenesis. In our study, β-tubulins (spots C24-1 and C24-2) with lower isoelectric points and higher phosphorylation levels were less abundant in the reproductive phase than in juvenile and adult vegetative phases. Second, the abundances of proteins exhibiting the same degree of phosphorylation differed significantly over the three ontogenetic phases. For example, all three Rubisco activase spots of group B38 were more abundant during the juvenile phase than during the other ontogenetic phases. As another example, the content of Rubisco large-chain spot 4791 of group A48 in seedling 02-18-081 was significantly lower in the reproductive phase than in juvenile or adult vegetative phases. These results consistently confirm that protein phosphorylation patterns changed across the three ontogenetic phases.

Phosphorylation of the Rubisco large subunit is critical for plant development. Light is the main factor affecting Rubisco phosphorylation, as phosphorylation of the Rubisco large subunit is light dependent [17]. Synthesis of the Rubisco large subunit, however, does not involve phosphorylation [18]. The physiologically active Rubisco large chains are usually phosphorylated on threonine and serine residues, while Rubisco small chains are typically phosphorylated on threonine and tyrosine residues. Dephosphorylation of these amino acids may cause dissociation of the large and small chains, resulting in decreased Rubisco activity [18,19]. In this study, juvenile-phase samples were collected from basal portions of seedlings, whereas reproductive-phase samples were taken from the upper canopy. Although the difference in leaf light exposure between samples might be expected to influence phosphorylation of the Rubisco large subunit, we detected no significant differences in net photosynthetic rate between ontogenetic phases (Additional file 10: Figure S6). The three groups of Rubisco large-subunit fragments (groups A6, A33 and A48) varied significantly with respect to protein abundance and degree of phosphorylation among ontogenetic phases. In our previous study, the abundance of 15 differentially expressed protein spots that were identical to the Rubisco large subunit or its segments differed drastically among developmental phases [16].

Phosphorylation of Rubisco activase is also very important for plant growth and development. Rubisco activase can promote the release of these analogs from the catalytic sites and maintain the enzyme in a catalytically active form. In the green alga *Chlamydomonas reinhardtii*, phosphorylation of Ser53 in Rubisco activase has been found to only occur in photosystem I [20]. Regulation of the ATPase activity of Rubisco activase determines the activation state of Rubisco, which responds to changes in stromal phosphorylation potential [21]. In all seedlings in this study, Rubisco activase spots were in greatest abundance during the juvenile phase, and then decreased. Similar dynamic changes have been detected previously in apple, with five out of six Rubisco activase spots in an earlier study expressed more actively in the juvenile phase [16]. ADP to ATP physiological ratios can significantly inhibit Rubisco activation in species containing both α- and β-Rubisco activase (e.g., *Arabidopsis* and *Camelina sativa*) or only β-Rubisco activase [e.g., tobacco (*Nicotiana tabacum*)]. In the *Arabidopsis* transformant rwt43, which expresses only β-Rubisco activase, Rubisco activase activity is insensitive to ADP [22]. The Rubisco activase detected in our study has not been further classified as α- or β-Rubisco activase.

Phosphorylation of tubulin is involved in the regulation of mitosis. In mitotic cells, phosphorylated tubulin is not conjugated with microtubules, but is instead present in soluble fractions and thus available for mitosis [23]. Reversible tyrosine phosphorylation is involved in the regulation of microtubule dynamics in plant cells, and tyrosine kinase inhibitors can delay mitosis [24]. The β-tubulin gene is an optimal reference gene because its expression is very stable in many species such as cut rose (*Rosa hybrida*) and sugar beet (*Beta vulgaris*) [25]. In our study, however, we found that tubulin phosphorylation levels were significantly different among ontogenetic phases. A higher abundance of more extensively phosphorylated β-tubulin spots having lower isoelectric points, i.e., spots C24-1 and C24-2, was observed during the juvenile phase; these elevated levels were most likely related to vigorous cell division during this ontogenetic phase. In leaves of apple seedlings, high levels of dihydrozeatin-type cytokinins have been observed during the juvenile phase [26], and the rice mutant *mori1*, defective for the juvenile to adult phase transition, maintains high cell-division activity in shoot apical meristem throughout its growth period [27]. Post-translational regulation is thus postulated to be responsible, at least partially, for decreased cell division activity during the vegetative phase change.

Protein phosphatase catalyzes dephosphorylation of phosphoserine, phosphothreonine, phosphotyrosine, and phosphohistidine [28]. Some spots, such as spot A33-3 and all spots in group A48, were still visible by Coomassie Brillant Blue R-350 staining after dephosphorylation with calf intestinal phosphatase. Similarly, upon treatment with highly efficient protein phosphatase followed by western blotting using an anti-phosphotyrosine antibody, very faint bands were still detectable in the sample [29]. Additionally, slight shifting of protein spot isoelectric points may occur after treatment with calf intestinal phosphatase. When proteins were treated with protein phosphatase and then separated by 2-DE over a broad pH range (pH 3–10), most detected phosphoproteins exhibited isoelectric point differences of nearly 0.05 pH unit [30].

Phosphorylation may also cause the same protein to appear with different molecular weights on 2-DE gels as a result of slight alterations in its molecular weight. For example, a phosphoprotein spot in one study was identified as a Rubisco large-chain subunit with an observed molecular mass of 25.4 kDa, a slight difference from its theoretical molecular mass of 26.5 kDa [31]. Additionally, Rubisco has been found with different molecular weights and isoelectric points on 2-DE gels [32,33]. In our previous study, 15 differentially expressed protein spots with varying molecular weights were identical to the Rubisco large subunit or its segments [16]. In the present experiment, the protein spots in group A with different molecular weights and isoelectric points were all identified as Rubisco large-subunit fragments. As seen on the gel image in Figure 2 (A33, panel a), the molecular weight of spot A33-1 seemed to be a slightly larger than that of A33-2 and A33-3; according to the MALDI-TOF-TOF mass spectrometry data, however, molecular weights of the three spots were identical.

## Conclusions

In this study, 55 spots of Rubisco large-chain fragments varied significantly in protein abundance and degree of phosphorylation among ontogenetic phases. The abundance of 27 Rubisco activase spots from juvenile to reproductive phases was characterized by a descending gradient. The abundance of more extensively phosphorylated β-tubulin spots with lower isoelectric points was higher in juvenile and adult vegetative phases. Results from Pro-Q Diamond phosphoprotein gel staining were consistent with those from calf intestinal phosphatase digestion proteomics, revealing that protein phosphorylation varied significantly among ontogenetic phases in apple seedlings. Most of these differences were consistent between seedlings and between hybrid populations. Our future work will focus on identifying the functions of these differentially phosphorylated proteins during different ontogenetic phases in apple seedlings.

## Methods

### Plant materials

Because an apple cultivar is actually a highly heterozygous genotype, cross hybrids segregate extensively. For our analysis of ontogenetic differences, we therefore simultaneously analyzed as biological replicates three seedlings derived from two hybrid apple populations: Jonathan × Golden Delicious (02-18-081 and 02-17-115) and Zisai Pearl × Red Fuji (07-07-133). Data generated using three seedlings was expected to be sufficiently robust to yield reliable results [34]. The Jonathan × Golden Delicious hybridization was performed in 2002; the seedlings were planted the following year at the Changli Institute of Pomology (Hebei, China), with fruiting beginning in 2007. Zisai Pearl was crossed with Red Fuji in 2007. The resulting seedlings were planted at China Agricultural University (Beijing, China) in 2008, with fruiting occurring in 2012. All seedlings were planted at a density of 0.5 m × 2.5 m and were subjected to conventional field management and pest control. A basal sprout and a relay shoot (i.e., an axillary shoot on which node numbers partially overlapped with those of the basal sprout) were allowed to grow from each seedling in early spring [16,35,36]. Fully expanded young leaves of identical shapes and sizes were sampled from juvenile, adult vegetative and reproductive parts of each plant in May 2011. The leaf samples were collected in three technical replicates (different leaves) from the same ontogenetic phase on the same plant. Beginning and ending nodes of juvenile, adult vegetative, and reproductive phases were located on the trunks of trees 02-18-081 and 02-17-115 using the criteria of our previous study [35] (Table 2). The corresponding nodes for 07-07-133 were not identified, and samples were instead collected using the estimation method described by Zhang et al. (2007) [36] (Table 2). Leaf samples were wrapped with aluminum foil, immersed immediately in liquid nitrogen, and stored in a -80°C freezer.

**Table 2:** **Sampled tree-trunk node numbers representing different ontogenetic phases in *Malus *** **hybrid seedlings**

**Seedling no.**	**J**	**V**	**R**
02-17-115	41-55	111-120	126-140
02-18-081	46-60	101-110	121-135
07-07-133	25-36	90-100	130-140

J, juvenile phase; V, adult vegetative phase; R, reproductive phase.

### Protein extraction

#### Trichloroacetic acid/acetone

Proteins were extracted by the trichloroacetic acid/acetone protocol [16]. Frozen leaves were fully ground in liquid nitrogen and transferred into three 2-mL Eppendorf tubes. Three volumes of extraction solution (10% trichloroacetic acid in acetone) were immediately added to the sample. After 30 s of vortexing, the mixture was left overnight at -20°C. The extraction mixture was centrifuged at 20,000 × *g* for 1 h at 4°C, and the supernatant was discarded. The pellet was resuspended, washed three times with ice-cold acetone, and centrifuged at 20,000 × *g* for 1 h at 4°C. The residue was then lyophilized under vacuum for 50 min. The dried powder was dissolved in 0.5 mL lysis buffer containing 7 M urea, 2 M thiourea, 4% (w/v) CHAPS, and 40 mM Tris-base, with the solution incubated at room temperature for 2 h to allow the sample to completely dissolve. Nucleic acid was eliminated by incubation in an ultrasonic bath for 5 min. The sample was then centrifuged at 4,000 × *g* for 60 min at 4°C, and the supernatant was collected. Protein concentrations were measured according to Bradford [37].

#### Alkaline phosphatase pre-treatment

Frozen leaves were fully ground in liquid nitrogen, mixed with extraction buffer [50 mM Tris–HCl (pH 8.4), 100 mM NaCl, 1 mM MgCl_2_, and 1 mM dithiothreitol (DTT)] excluding phosphatase inhibitors, and incubated with calf intestinal phosphatase (Promega, Madison, WI, USA) or phosphatase buffer alone at 37°C for 2 h [31]. Three volumes of extraction solution (10% trichloroacetic acid in acetone) was immediately added. After 30 s of vortexing, the mixture was left overnight at -20°C, with subsequent steps identical to those of the procedure in the previous paragraph.

### Two-dimensional electrophoresis (2-DE)

Isoelectrofocusing (IEF) of proteins was performed on an Ettan IPGphor 3 IEF system (GE Healthcare, Piscataway, NJ, USA) using linear IPG strips (pH 3–10, 24 cm; GE Healthcare, Piscataway, NJ, USA).The amount of loaded protein was 500 μg per gel. Sample volumes were adjusted to 450 μL with rehydration solution, and samples were incubated in an IPGbox (GE Healthcare) for 12 h. IEF was carried out at a constant current of 50 mA per strip using the following program: 500 V for 1 h, 5,000 V for 1 h, 8,000 V for 2 h, 8000 V 55,000 Vh, and 2,500 V for 10 h. After IEF, the IPG strips were removed from the holder and equilibrated with sodium dodecyl sulfate (SDS) equilibration buffer [0.05 M Tris–HCl (pH 8.8), 6 M urea, 30% (v/v) glycerol, 2% (w/v) SDS, 1% DTT, and a trace of bromophenol blue] for 15 min, followed by a second equilibration with the same volume of equilibration buffer including 4% iodoacetamide/SDS, but without DTT, for 15 min.

The equilibrated strips were placed on the top of 12% SDS-polyacrylamide gels and sealed with 0.2% (w/v) agarose with a trace of bromophenol blue. The gels were run in parallel with a Peltier-cooled EttanDaltTwelve vertical electrophoresis system (GE Healthcare) at 2 W/strip for 30 min, followed by 15 W/strip for 5–6 h until the bromophenol blue reached the bottom of the gel.

Twenty-seven gels were prepared per seedling. A set of nine gels (3 ontogenetic phases × 3 technical replicates) containing global proteins was stained with Coomassie Brilliant Blue R-350. Another nine gels, also containing global proteins, were stained with Pro-Q Diamond, while nine gels loaded with dephosphorylated proteins pre-treated with calf intestinal phosphatase were stained with Coomassie Brilliant Blue R-350.

### 2-DE gel staining and image analysis

For Pro-Q Diamond phosphoprotein staining, the gels were placed in a polypropylene plastic box and fixed in fixation solution (50% [v/v] methanol and 10% [v/v] acetic acid) at room temperature with gentle agitation. After 1 h, the solution was replaced with fresh solution, and incubation was allowed to continue overnight. The gels were then washed three times for 20 min with Milli-Q water, and placed for 1 h in the dark in 500 mL Pro-Q Diamond staining solution (Molecular Probes, Carlsbad, CA, USA). After staining, the gels were washed three times in destaining solution [50 mM sodium acetate (pH 4.0) and 20% acetonitrile] for 30 min. The gels were then washed three times for 10 min with Milli-Q water and scanned with an FLA-9000 ImageReader (Fujifilm, Tokyo, Japan). Gels not subjected to Pro-Q Diamond staining were stained with PhastGel R-350 Coomassie Brilliant Blue tablets (GE Healthcare) and scanned with an ImageScanner III (GE Healthcare). Images of 2-DE gels stained with either Pro-Q Diamond or Coomassie Brilliant Blue were analyzed using ImageMaster 2-D platinum 7.0 software (GE Healthcare). During the image analysis, corresponding protein spots on gel images of global proteins extracted with trichloroacetic acid/acetone and dephosphorylated proteins extracted with calf intestinal phosphatase were aligned and matched to each other, and their volumes were quantified. Optimized image parameters were as follows: Smooth 2, Saliency 50, and Min Area 5. The gel images were normalized according to the total quantity in the analysis set. This normalization method provided by ImageMaster 2-D platinum 7.0 software divided each spot abundance value by the sum of total spot abundance values to obtain individual relative spot abundance. The signal value of phosphoprotein spots on images of 2-DE gels stained by Pro-Q Diamond was quantified using ImageMaster 2-D platinum 7.0. The phosphoprotein spots were manually aligned and matched to the corresponding spots of global proteins and dephosphorylated proteins according to their molecular weights and isoelectric points. Student’s *t*-test was used to evaluate significance of expression abundance differences between protein spots at the *p* < 0.05 level.

### Identification of proteins

Differentially expressed protein spots were selected from gels of global proteins extracted with trichloroacetic acid/acetone, and the phosphorylation was validated by Pro-Q Diamond phosphoprotein-selective staining and by comparison with dephosphorylated 2-DE gel spots from the three seedlings at the three ontogenetic phases. The spots were manually excised and destained until colorless with 100 mL of 50% acetonitrile in 50 mM ammonium bicarbonate for at least 1 h. After a brief centrifugal spin, the supernatant was removed, and the residue was re-suspended in acetonitrile. After centrifuging, the supernatant was discarded and the residue was vacuum-dried for 1 h. Trypsin (2 mL, 50 ng/mL) was added to the sample. The mixture was held at 4°C for 60 min, and then incubated in a water bath at 37°C for 2 h. Tryptic peptide (1 mL) was deposited onto the MALDI target and analyzed with an AB4800 MALDI-TOF-TOF mass spectrometer (Applied Biosystems, Framingham, MA, USA). The software program 4000 Series Explorer Version 3.0 (Applied Biosystems) was used to create output files. Protein spots were compared against the SwissProt database (http://web.expasy.org/docs/swiss-prot_guideline.html) using the Mascot protein search engine (Matrix Science, London, UK). Any protein spots remaining unidentified were searched against the NCBI nr database (National Center for Biotechnology Information; http://www.ncbi.nlm.nih.gov). Typical search parameters were as follows: mass accuracy = ± 0.3 Da; allowed number of missed cleavage sites for each search = 1; enzyme = trypsin; fixed modifications = Carbamidomethyl (C); variable modifications = Oxidation (M) and Phosphorylation (STY); taxonomy = Viridiplantae (Green Plants). Only proteins with CIs ≥ 95% were considered to be validly identified.

## Competing interests

The authors declare that they have no competing interests.

## Authors’ contributions

Yan Wang designed and carried out experiments, analyzed protein data, and helped write the manuscript. Yi Wang, YBZ and DMC participated in the experiments. ZHH supervised the experimental design. XZZ conceived the proposed study, designed the experiments, analyzed the data, and helped write the manuscript. All authors read and approved the final manuscript.

## Supplementary Material

Additional file 1: Figure S1 2-DE images of differentially expressed phosphorylated proteins in different ontogenetic phases of apple seedling 02-17-115. The seedling 02-17-115 was derived from a cross hybrid ‘Jonathan’ × ‘Golden Delicious’, the proteins were extracted from leaf sample. Juvenile, adult vegetative and reproductive phases are designated by J, V and R, respectively. Proteins were profiled in the first dimension by isoelectrofocusing using linear IPG stripes (pH 3–10, 24 cm) and on SDS-polyacrylamide gels in the second dimension. Phosphorylated protein groups are indicated by open squares. Panel a depicts global proteins stained with Coomassie Brilliant Blue R-350, panel b shows total phosphorylated proteins stained with Pro-Q Diamond, and panel c corresponds to dephosphorylated proteins pre-treated with calf intestinal phosphatase and stained with Coomassie Brilliant Blue R-350. Approximate molecular masses and isoelectric points are indicated on the right edge and top margin, respectively. Click here for file

Additional file 2: Figure S2 2-DE images of differentially expressed phosphorylated proteins in different ontogenetic phases of apple seedling 07-07-133. The seedling 07-07-133 was derived from a cross hybrid ‘Zisai Pearl’ × ‘Red Fuji’, the proteins were extracted from leaf sample. Juvenile, adult vegetative and reproductive phases are designated by J, V and R, respectively. Proteins were profiled in the first dimension by isoelectrofocusing using linear IPG stripes (pH 3–10, 24 cm) and on SDS-polyacrylamide gels in the second dimension. Phosphorylated protein groups are indicated by open squares. Panel a represents global proteins stained with Coomassie Brilliant Blue R-350; panel b indicates total phosphorylated proteins stained with Pro-Q Diamond, and panel c shows dephosphorylated proteins pre-treated with calf intestinal phosphatase and stained with Coomassie Brilliant Blue R-350. Approximate molecular masses and isoelectric points are indicated on the right edge and top margin, respectively. Click here for file

Additional file 3: Table S1 Relative spot abundances at different ontogenetic phases of three apple seedlings. J, V and R correspond to juvenile, adult vegetative and reproductive phases, respectively. Each spot number represents the group number and the spot’s gel position. For example, A33-1 refers to the first spot (from left to right) of group A33. Click here for file

Additional file 4: Table S2 Student’s *t*-test of expression in corresponding spots among different ontogenetic phases in three apple seedlings. J, V and R designate juvenile, adult vegetative and reproductive phases, respectively. Data were from two-dimensional electrophoretic gels containing global proteins. Statistical analysis using Student’s *t*-test at the *p* < 0.05 level was used to assess the significance of expression abundance differences among the three phases. Each spot number represents the group number and the spot’s gel position. For example, A33-1 refers to the first spot (from left to right) of group A33. Click here for file

Additional file 5: Table S3 Putative phosphosites identified by MALDI-TOF-TOF from different ontogenetic phases of three apple seedlings. J, V and R correspond to juvenile, adult vegetative and reproductive phases, respectively. Images of 02-18-081, 02-17-115 and 07-07-133 are shown in Figures 1, 2 and Figures S1, S2, S4, S5. Click here for file

Additional file 6: Figure S3 Annotated spectra of protein spots successfully identified in three apple seedlings. Images of 02-18-081, 02-17-115 and 07-07-133 are shown in Figures 1 and 2 and Figures S1, S2, S4, S5. Click here for file

Additional file 7: Table S4 Group D identified by MALDI-TOF-TOF from different ontogenetic phases of three apple seedlings. J, V and R correspond to juvenile, adult vegetative and reproductive phases, respectively. Images of 02-18-081, 02-17-115 and 07-07-133 are shown in Figure 1, S1 and S2. Each spot number represents the group number and the spot’s gel position. For example, D1-1 refers to the first spot (from left to right) of group D1. Click here for file

Additional file 8: Figure S4 Zoomed-in images of phosphorylated protein in different ontogenetic phases of apple seedling 02-17-115. The seedling 02-17-115 was derived from a cross hybrid ‘Jonathan’ × ‘Golden Delicious’, the proteins were extracted from leaf sample. Juvenile, adult vegetative and reproductive phases are designated by J, V and R, respectively. Panel a shows global proteins stained with Coomassie Brilliant Blue R-350, panel b corresponds to total phosphorylated proteins stained with Pro-Q Diamond, and panel c represents dephosphorylated proteins pre-treated with calf intestinal phosphatase and stained with Coomassie Brilliant Blue R-350. In this figure, spot numbers referenced in the text and in Table 1 (with their corresponding spots in parentheses) are A6-1 (spots 679/1085/811 in panel a; 313/390/402 in panel b), A33-1 (not visible in this seedling), A33-2 (-/1088/- in panel a; not visible in panel b), A33-3 (542/887/694 in panel a; 387/407/421 in panel b), A48-1 (694/1091/- in panel a; 332/419/432 in panel b), A48-2 (696/1095/814 in panel a; not visible in panel b), A48-3 (697/1097/721 in panel a; 331/417/431 in panel b), B38-1 (686/507/341 in panel a; 380/464/458 in panel b), B38-2 (350/509/342 in panel a; 382/465/460 in panel b), B38-3 (687/527/336 in panel a; 384/466/463 in panel b), C24-1 (683/417/243 in panel a; 377/461/450 in panel b), C24-2 (284/1084/247 in panel a; 171/462/189 in panel b), and C24-3 (285/422/249 in panel a; 173/463/199 in panel b). Click here for file

Additional file 9: Figure S5 Zoomed-in images of phosphorylated protein in different ontogenetic phases of apple seedling 07-07-133. The seedling 07-07-133 was derived from a cross hybrid ‘Zisai Pearl’ × ‘Red Fuji’, the proteins were extracted from leaf sample. Juvenile, adult vegetative and reproductive phases are designated by J, V and R, respectively. Panel a represents global proteins stained with Coomassie Brilliant Blue R-350, panel b shows total phosphorylated proteins stained with Pro-Q Diamond, and panel c corresponds to dephosphorylated proteins pre-treated with calf intestinal phosphatase and stained with Coomassie Brilliant Blue R-350. In this figure, spot numbers referenced in the text and in Table 1, with their corresponding spots in parentheses, are A6-1 (spots 420/302/312 in panel a; 837/744/5819 in panel b), A33-1 (345/245/182 in panel a; 708/603/5626 in panel b), A33-2 (348/247/183 in panel a; 711/604/5632 in panel b), A33-3 (346/244/180 in panel a; not visible in panel b), A48-1 (428/293/325 in panel a; 727/624/5822 in panel b), A48-2 (433/294/326 in panel a; 725/623/5823 in panel b), A48-3 (435/295/327 in panel a; 721/749/5826 in panel b), B38-1 (132/72/53 in panel a; 468/280/5821 in panel b), B38-2 (127/71/50 in panel a; 469/281/5144 in panel b), B38-3 (124/69/49 in panel a; 466/283/5139 in panel b), C24-1 (422/304/- in panel a; not visible in panel b), C24-2 425/306/- in panel a; -/234/- in panel b), and C24-3 (426/47/316 in panel a; 397/236/5083 in panel b). Click here for file

Additional file 10: Figure S6 Net photosynthetic rate of three apple seedlings of ‘Zisai Pearl’ × ‘Red Fuji’. Net photosynthetic rate was measured in leaves at different positional levels on ‘Zisai Pearl’ × ‘Red Fuji’ hybrid seedlings (A: 07-07-115; B: 07-07-133 and C: 07-09-141). ‘Zisai Pearl’ was crossed with ‘Red Fuji’ in 2007. The resulting seedlings were planted at China Agricultural University (Beijing, China) in 2008, with fruiting occurring in 2012. All seedlings were planted at a density of 0.5 m × 2.5 m and were subjected to conventional field management and pest control. Net photosynthetic rate was measured with a LI 6400 photosynthetic system (LI-COR, Lincoln, NE, USA). Net photosynthetic rate of three randomly selected leaves per seedling was measured every 10 nodes, from the 20th to the 150th node, on May 15–17, 2012, in the morning. Net photosynthetic rate did not increase with the node, demonstrating that leaf light perception was not related to leaf position. Click here for file
