# Supplementary figures and images for: Protein phosphorylation differs significantly among ontogenetic phases in Malus seedlings
Source: Proteome Sci. 2014 May 25;12:31. doi: 10.1186/1477-5956-12-31 (PMC4046019; doi:10.1186/1477-5956-12-31)

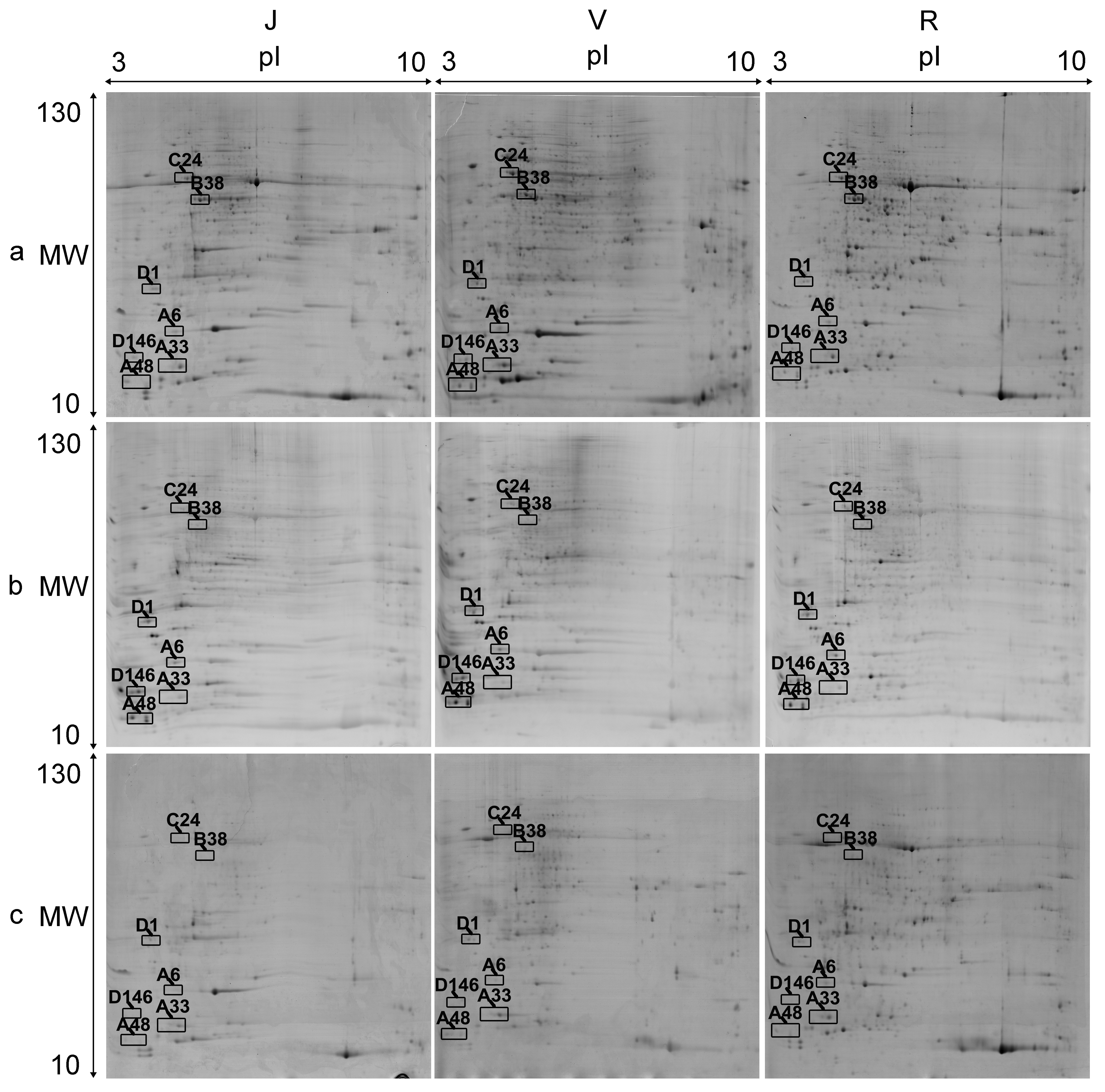

Supplement: Additional file 1: Figure S1 — 2-DE images of differentially expressed phosphorylated proteins in different ontogenetic phases of apple seedling 02-17-115. The seedling 02-17-115 was derived from a cross hybrid ‘Jonathan’ × ‘Golden Delicious’, the proteins were extracted from leaf sample. Juvenile, adult vegetative and reproductive phases are designated by J, V and R, respectively. Proteins were profiled in the first dimension by isoelectrofocusing using linear IPG stripes (pH 3–10, 24 cm) and on SDS-polyacrylamide gels in the second dimension. Phosphorylated protein groups are indicated by open squares. Panel a depicts global proteins stained with Coomassie Brilliant Blue R-350, panel b shows total phosphorylated proteins stained with Pro-Q Diamond, and panel c corresponds to dephosphorylated proteins pre-treated with calf intestinal phosphatase and stained with Coomassie Brilliant Blue R-350. Approximate molecular masses and isoelectric points are indicated on the right edge and top margin, respectively. [file 1477-5956-12-31-S1.tiff]

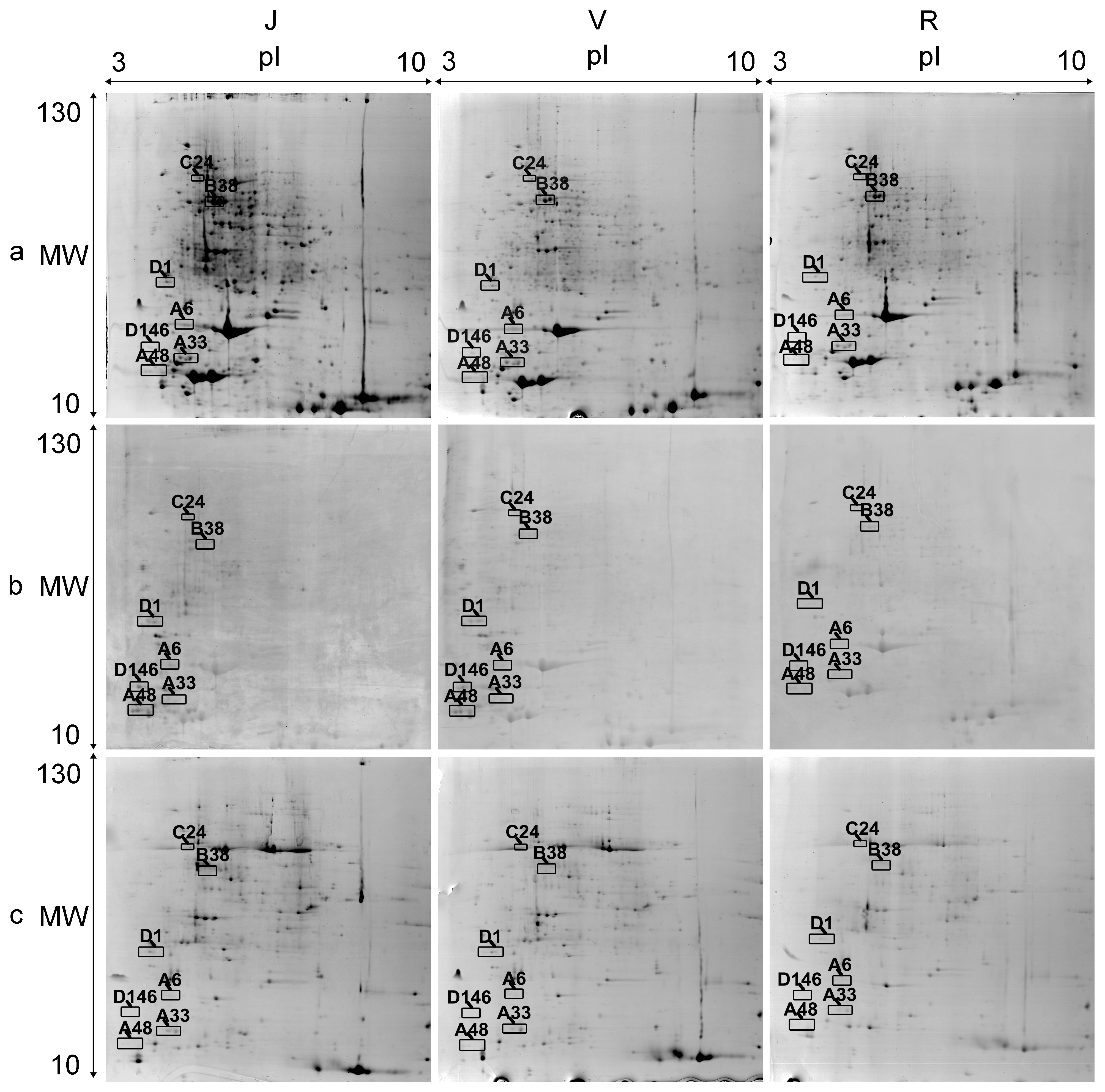

Supplement: Additional file 2: Figure S2 — 2-DE images of differentially expressed phosphorylated proteins in different ontogenetic phases of apple seedling 07-07-133. The seedling 07-07-133 was derived from a cross hybrid ‘Zisai Pearl’ × ‘Red Fuji’, the proteins were extracted from leaf sample. Juvenile, adult vegetative and reproductive phases are designated by J, V and R, respectively. Proteins were profiled in the first dimension by isoelectrofocusing using linear IPG stripes (pH 3–10, 24 cm) and on SDS-polyacrylamide gels in the second dimension. Phosphorylated protein groups are indicated by open squares. Panel a represents global proteins stained with Coomassie Brilliant Blue R-350; panel b indicates total phosphorylated proteins stained with Pro-Q Diamond, and panel c shows dephosphorylated proteins pre-treated with calf intestinal phosphatase and stained with Coomassie Brilliant Blue R-350. Approximate molecular masses and isoelectric points are indicated on the right edge and top margin, respectively. [file 1477-5956-12-31-S2.tif]

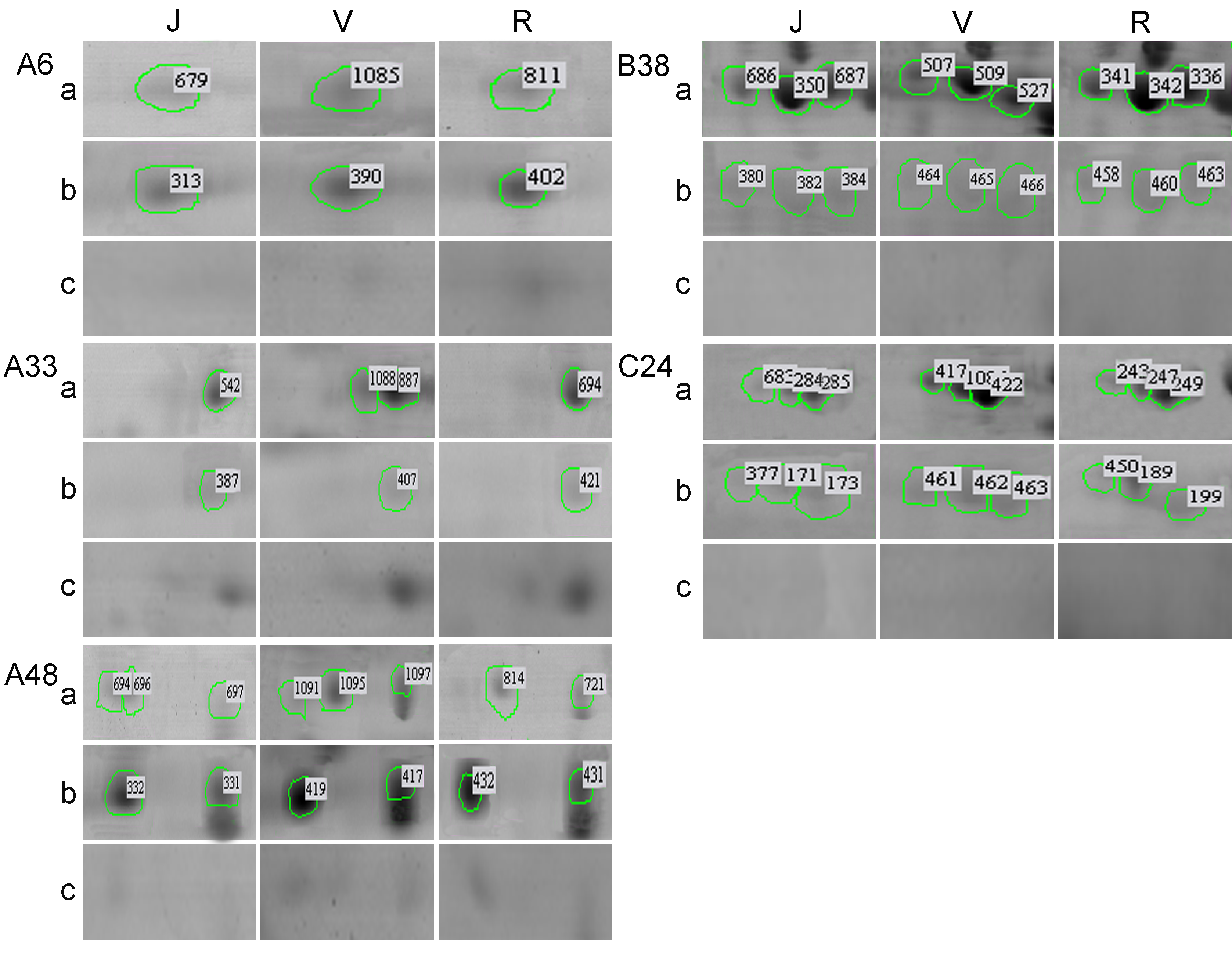

Supplement: Additional file 8: Figure S4 — Zoomed-in images of phosphorylated protein in different ontogenetic phases of apple seedling 02-17-115. The seedling 02-17-115 was derived from a cross hybrid ‘Jonathan’ × ‘Golden Delicious’, the proteins were extracted from leaf sample. Juvenile, adult vegetative and reproductive phases are designated by J, V and R, respectively. Panel a shows global proteins stained with Coomassie Brilliant Blue R-350, panel b corresponds to total phosphorylated proteins stained with Pro-Q Diamond, and panel c represents dephosphorylated proteins pre-treated with calf intestinal phosphatase and stained with Coomassie Brilliant Blue R-350. In this figure, spot numbers referenced in the text and in Table 1 (with their corresponding spots in parentheses) are A6-1 (spots 679/1085/811 in panel a; 313/390/402 in panel b), A33-1 (not visible in this seedling), A33-2 (-/1088/- in panel a; not visible in panel b), A33-3 (542/887/694 in panel a; 387/407/421 in panel b), A48-1 (694/1091/- in panel a; 332/419/432 in panel b), A48-2 (696/1095/814 in panel a; not visible in panel b), A48-3 (697/1097/721 in panel a; 331/417/431 in panel b), B38-1 (686/507/341 in panel a; 380/464/458 in panel b), B38-2 (350/509/342 in panel a; 382/465/460 in panel b), B38-3 (687/527/336 in panel a; 384/466/463 in panel b), C24-1 (683/417/243 in panel a; 377/461/450 in panel b), C24-2 (284/1084/247 in panel a; 171/462/189 in panel b), and C24-3 (285/422/249 in panel a; 173/463/199 in panel b). [file 1477-5956-12-31-S8.tif]

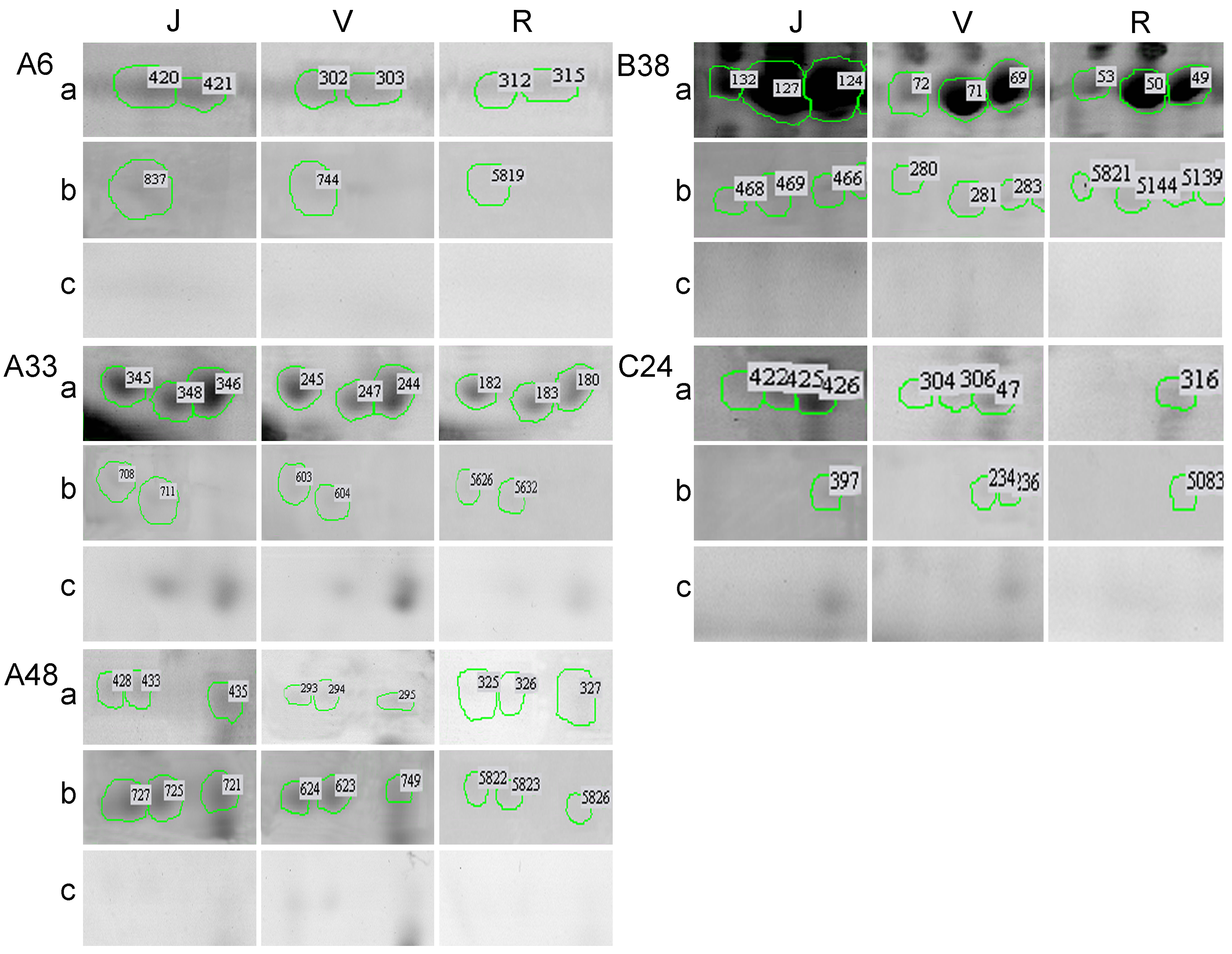

Supplement: Additional file 9: Figure S5 — Zoomed-in images of phosphorylated protein in different ontogenetic phases of apple seedling 07-07-133. The seedling 07-07-133 was derived from a cross hybrid ‘Zisai Pearl’ × ‘Red Fuji’, the proteins were extracted from leaf sample. Juvenile, adult vegetative and reproductive phases are designated by J, V and R, respectively. Panel a represents global proteins stained with Coomassie Brilliant Blue R-350, panel b shows total phosphorylated proteins stained with Pro-Q Diamond, and panel c corresponds to dephosphorylated proteins pre-treated with calf intestinal phosphatase and stained with Coomassie Brilliant Blue R-350. In this figure, spot numbers referenced in the text and in Table 1, with their corresponding spots in parentheses, are A6-1 (spots 420/302/312 in panel a; 837/744/5819 in panel b), A33-1 (345/245/182 in panel a; 708/603/5626 in panel b), A33-2 (348/247/183 in panel a; 711/604/5632 in panel b), A33-3 (346/244/180 in panel a; not visible in panel b), A48-1 (428/293/325 in panel a; 727/624/5822 in panel b), A48-2 (433/294/326 in panel a; 725/623/5823 in panel b), A48-3 (435/295/327 in panel a; 721/749/5826 in panel b), B38-1 (132/72/53 in panel a; 468/280/5821 in panel b), B38-2 (127/71/50 in panel a; 469/281/5144 in panel b), B38-3 (124/69/49 in panel a; 466/283/5139 in panel b), C24-1 (422/304/- in panel a; not visible in panel b), C24-2 425/306/- in panel a; -/234/- in panel b), and C24-3 (426/47/316 in panel a; 397/236/5083 in panel b). [file 1477-5956-12-31-S9.tif]

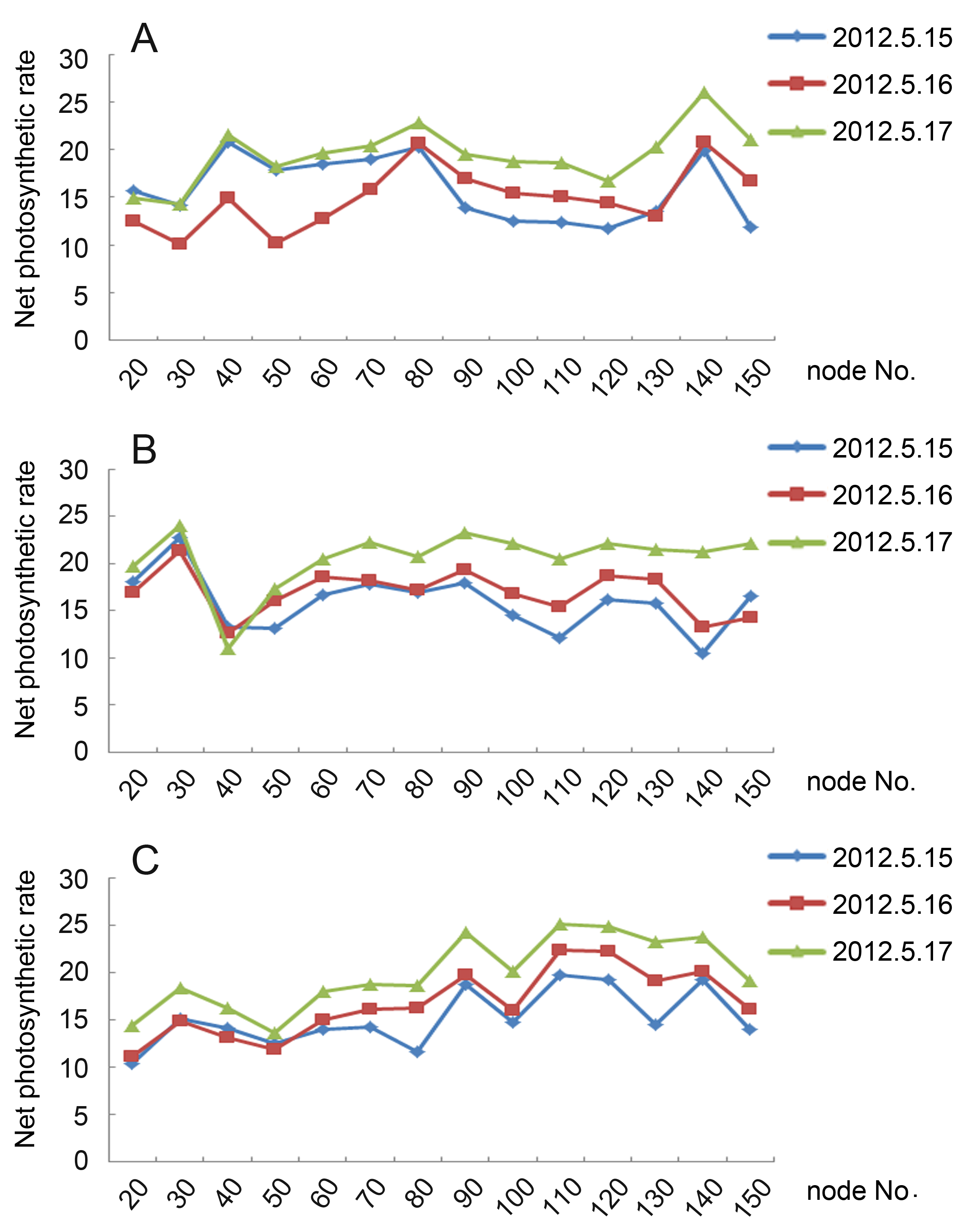

Supplement: Additional file 10: Figure S6 — Net photosynthetic rate of three apple seedlings of ‘Zisai Pearl’ × ‘Red Fuji’. Net photosynthetic rate was measured in leaves at different positional levels on ‘Zisai Pearl’ × ‘Red Fuji’ hybrid seedlings (A: 07-07-115; B: 07-07-133 and C: 07-09-141). ‘Zisai Pearl’ was crossed with ‘Red Fuji’ in 2007. The resulting seedlings were planted at China Agricultural University (Beijing, China) in 2008, with fruiting occurring in 2012. All seedlings were planted at a density of 0.5 m × 2.5 m and were subjected to conventional field management and pest control. Net photosynthetic rate was measured with a LI 6400 photosynthetic system (LI-COR, Lincoln, NE, USA). Net photosynthetic rate of three randomly selected leaves per seedling was measured every 10 nodes, from the 20th to the 150th node, on May 15–17, 2012, in the morning. Net photosynthetic rate did not increase with the node, demonstrating that leaf light perception was not related to leaf position. [file 1477-5956-12-31-S10.tif]
