# Supplementary material for: Protein phosphorylation differs significantly among ontogenetic phases in Malus seedlings
Source: Proteome Sci. 2014 May 25;12:31. doi: 10.1186/1477-5956-12-31 (PMC4046019; doi:10.1186/1477-5956-12-31)
Supplement: Additional file 6: Figure S3 — Annotated spectra of protein spots successfully identified in three apple seedlings. Images of 02-18-081, 02-17-115 and 07-07-133 are shown in Figures 1 and 2 and Figures S1, S2, S4, S5. [file 1477-5956-12-31-S6.pdf]

Figure S3 Annotated spectra of protein spots successfully identified in three apple seedlings.

02-18-081:

A6:

Spot 5298:

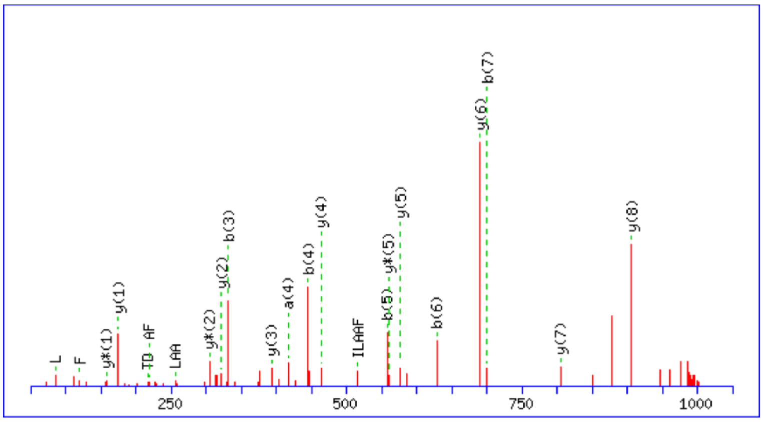

Spot 4227:

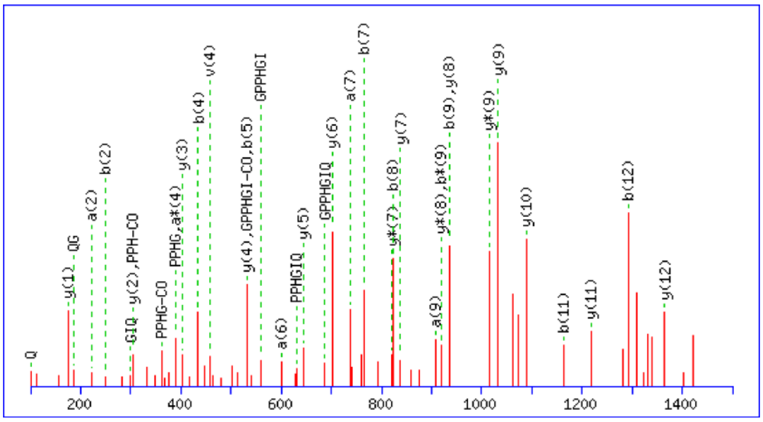

Spot 4965:

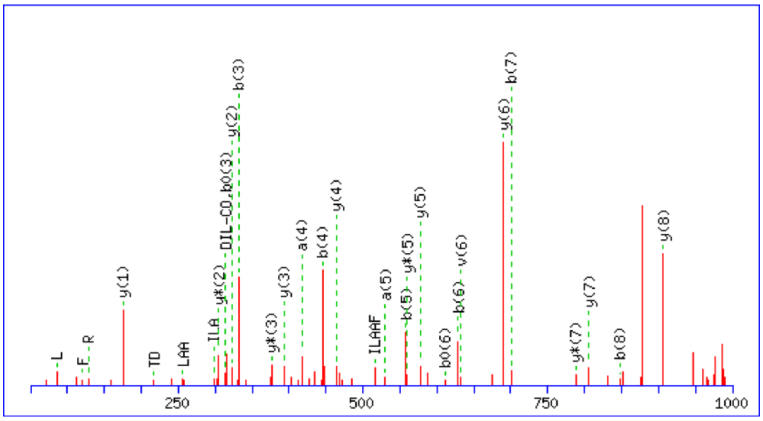

A33:

Spot 4994:

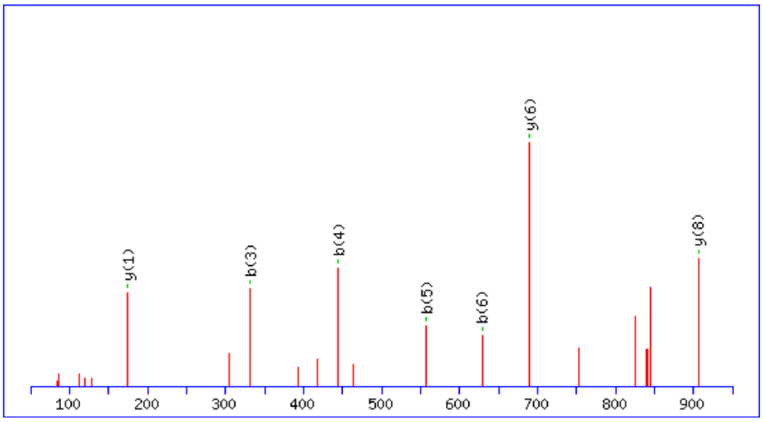

Spot 4998:

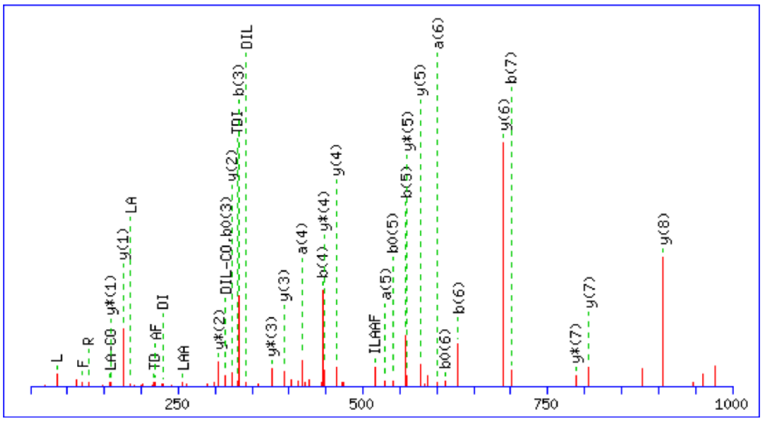

Spot 4996:

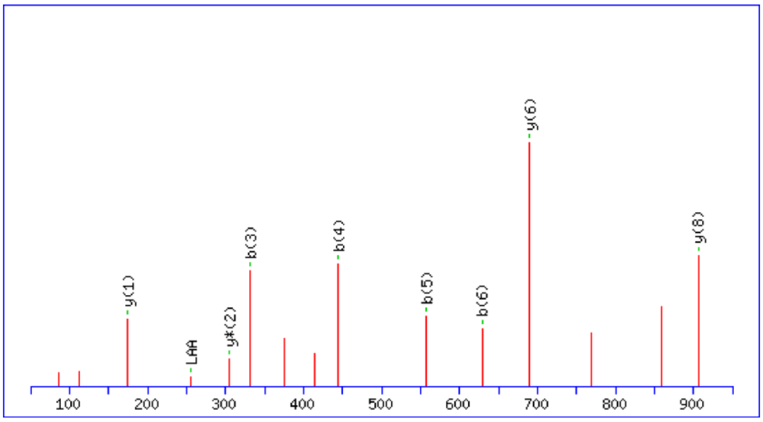

Spot 3929:

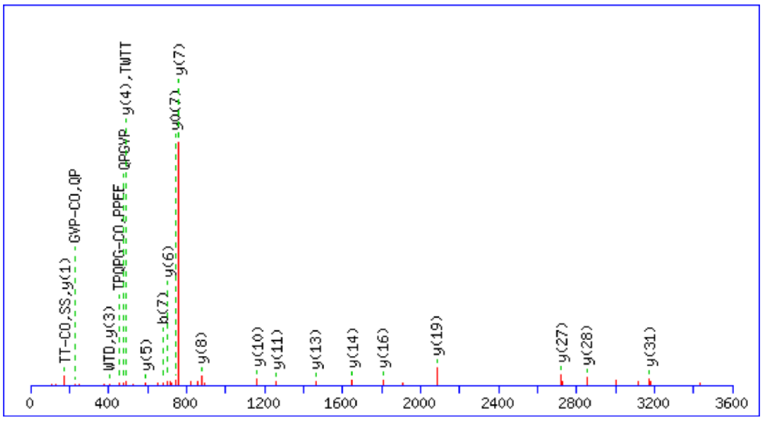

Spot 3931：

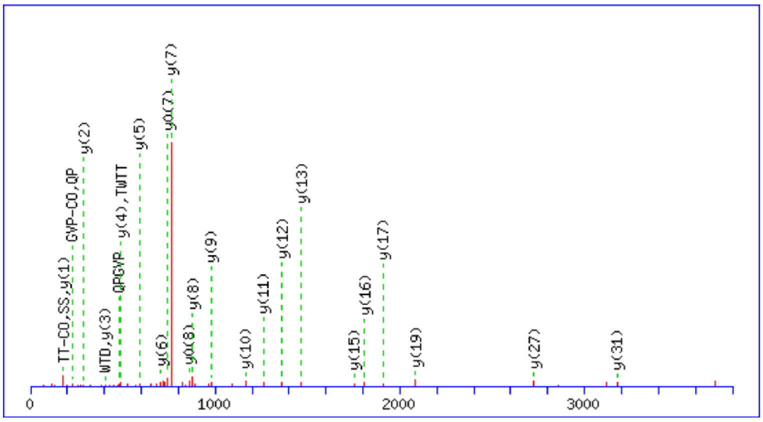

Spot 3927：

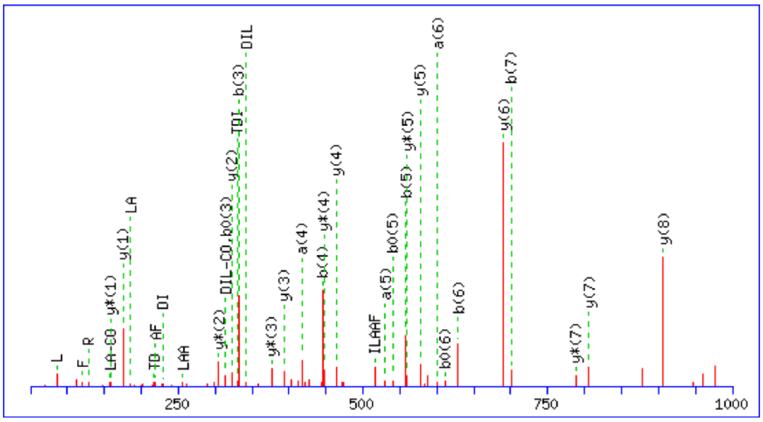

Spot 4779：

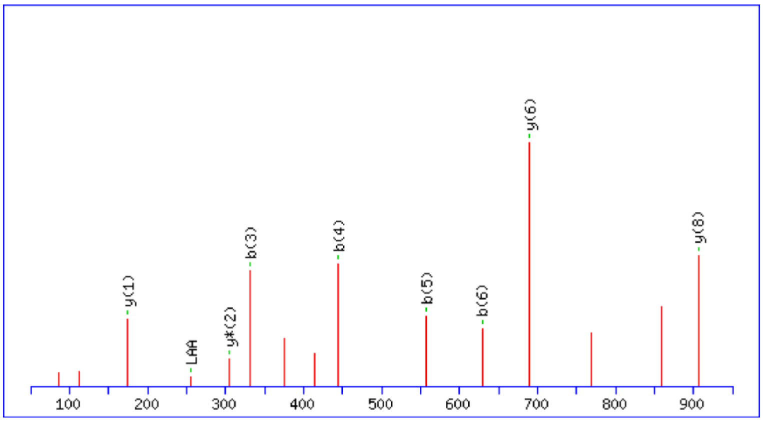

A48:

Spot 5026：

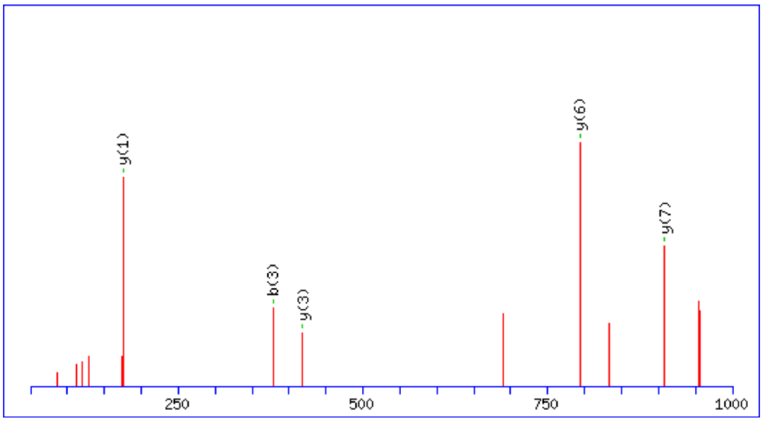

Spot 5022：

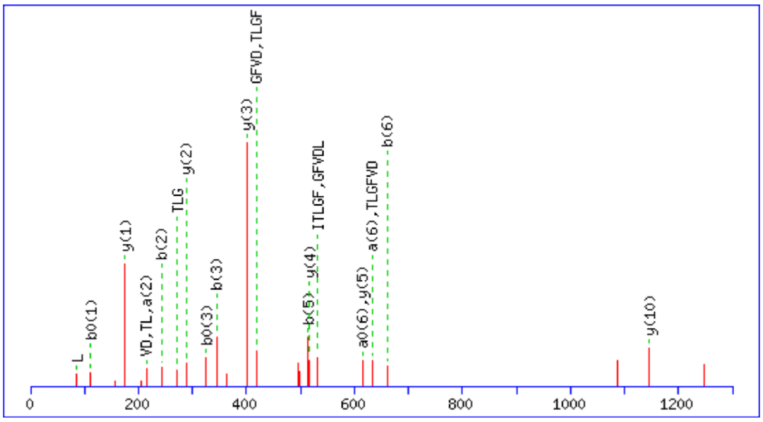

Spot 5027：

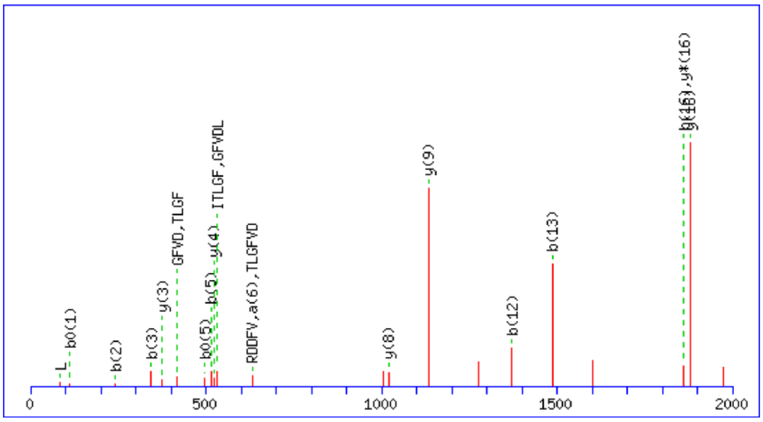

Spot 4234：

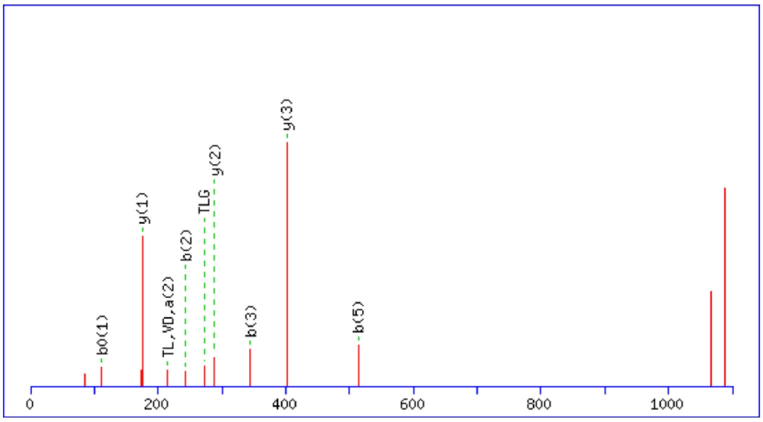

Spot 4236：

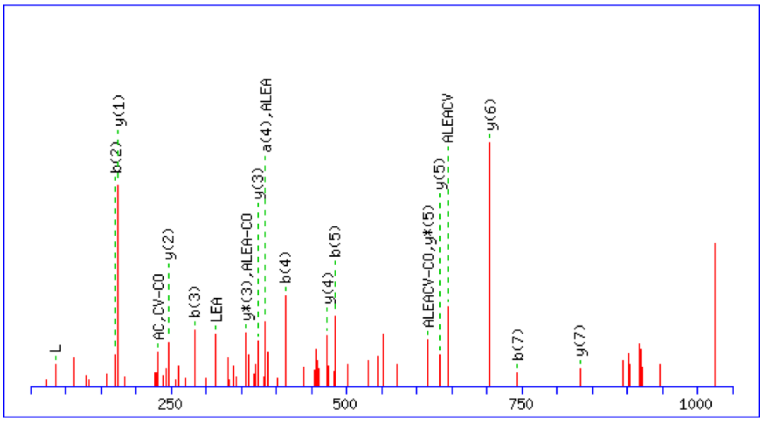

Spot 4238：

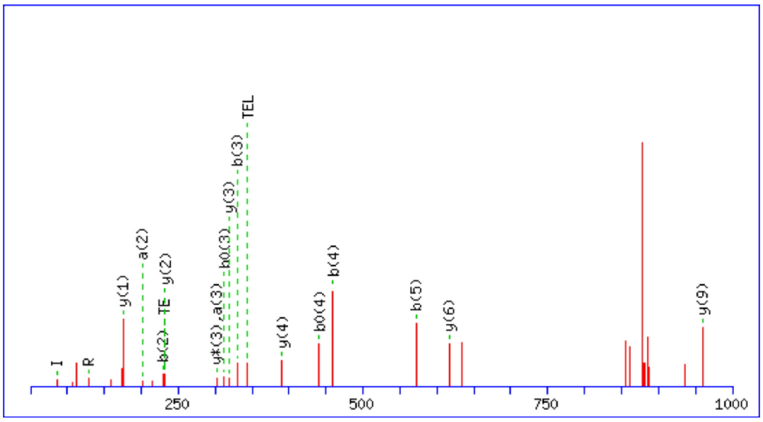

Spot 4791：

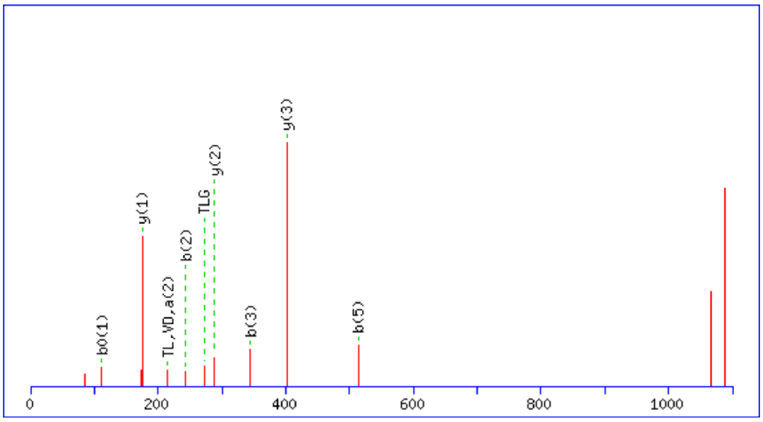

Spot 4792：

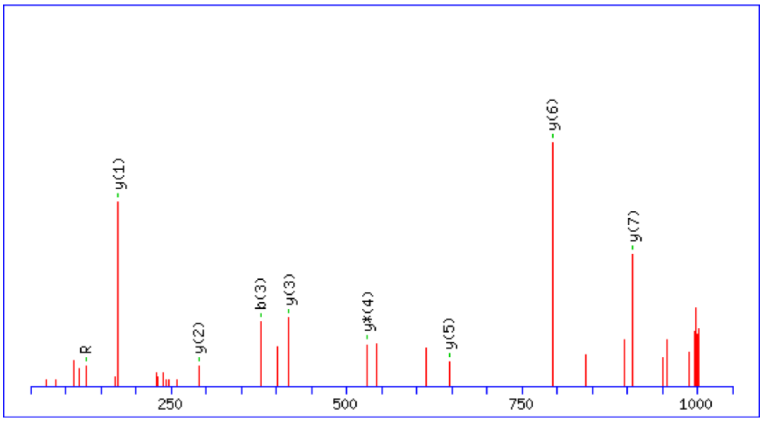

Spot 4796：

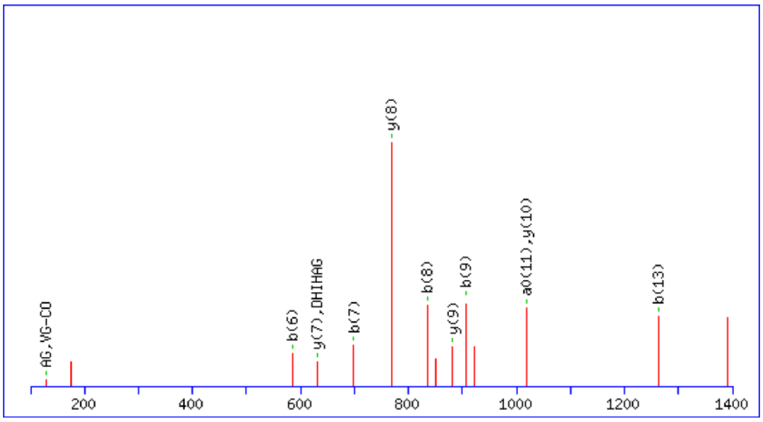

B38:

Spot 4539:

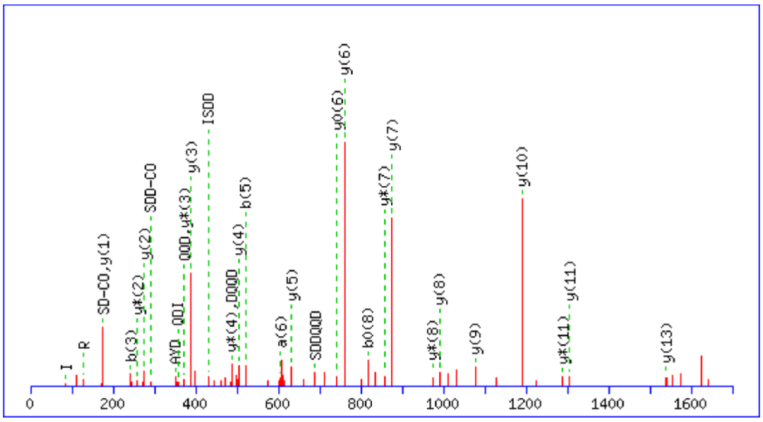

Spot 4540:

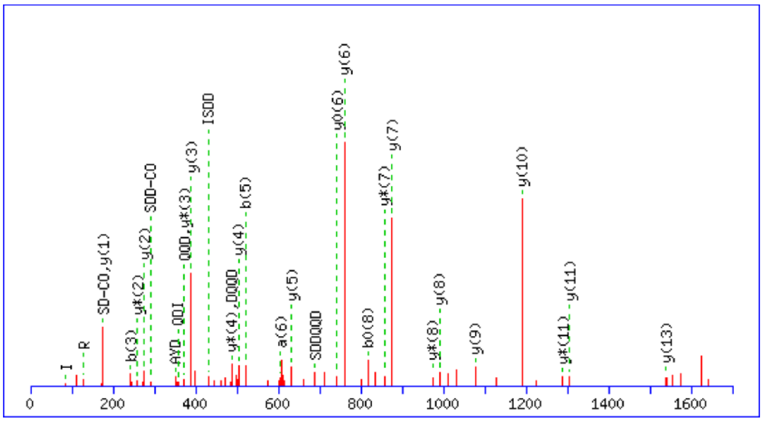

Spot 4546:

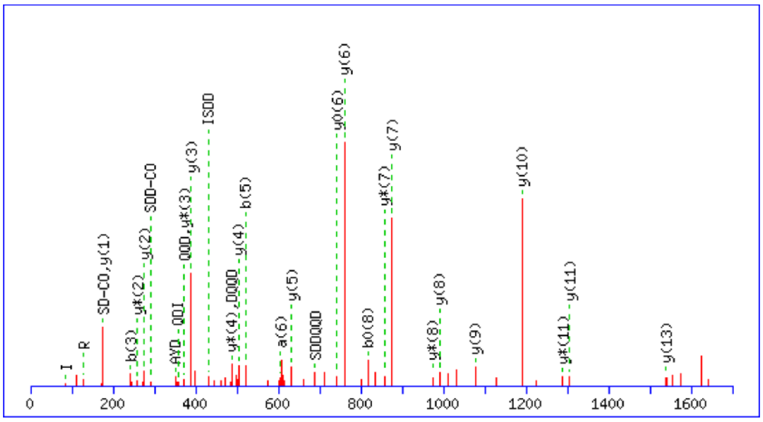

Spot 3595:

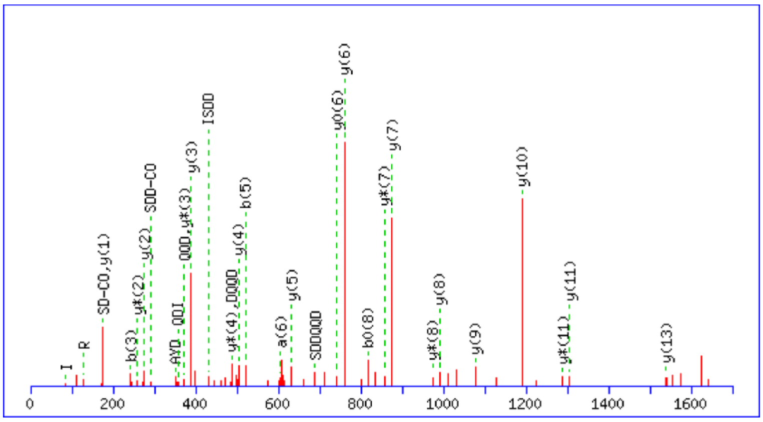

Spot 3596:

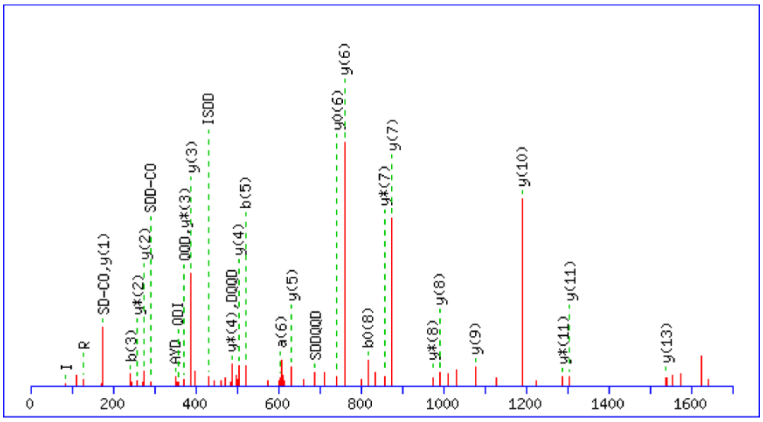

Spot 3593:

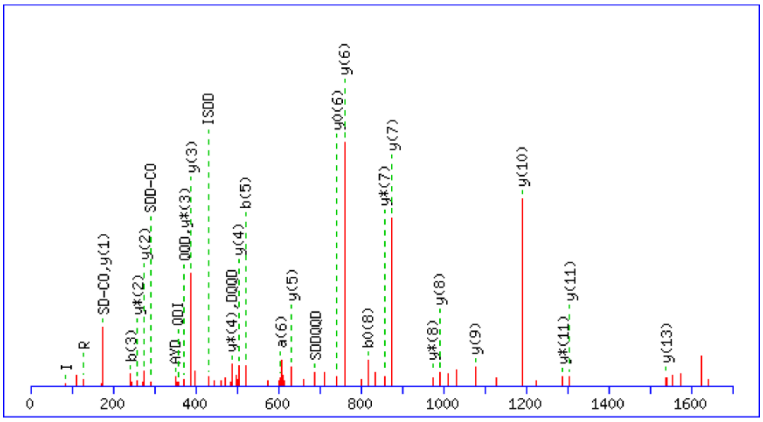

Spot 4528:

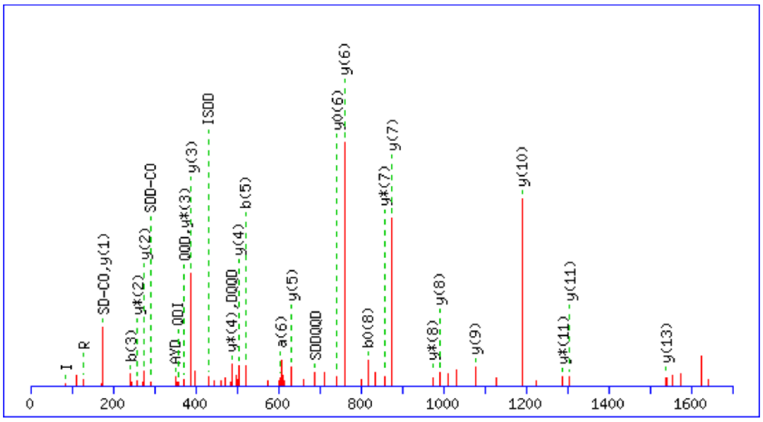

Spot 4529:

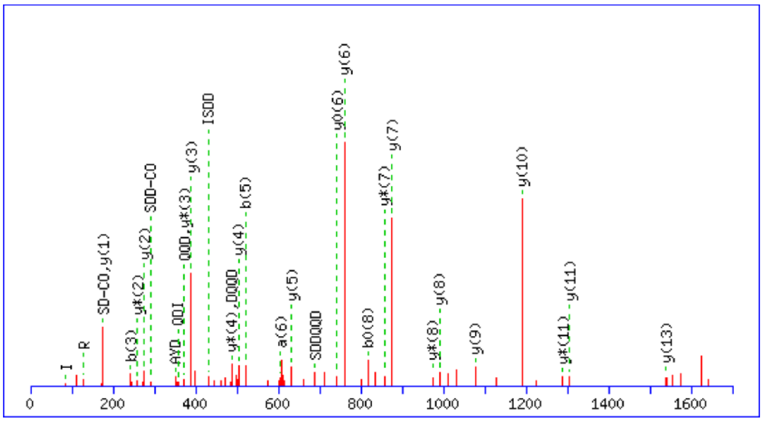

Spot 4531:

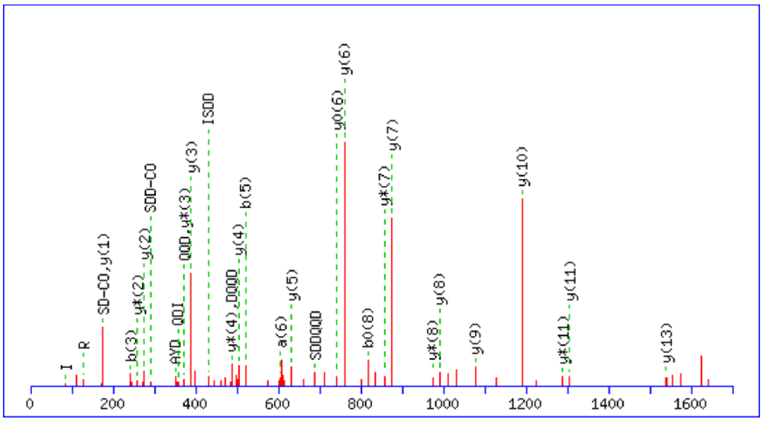

C24:

Spot 4471:

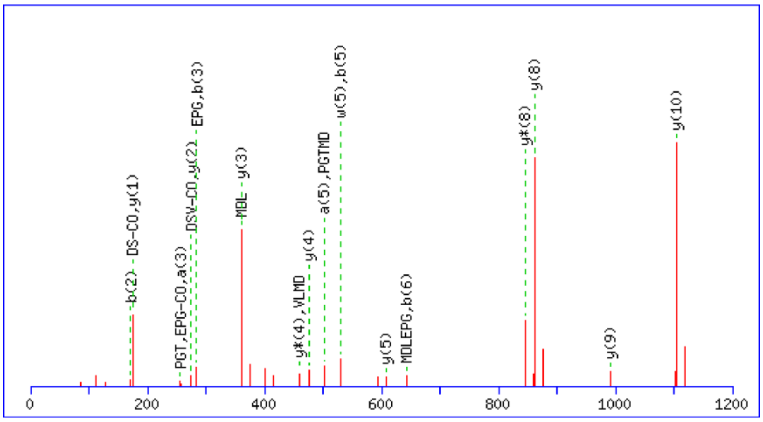

Spot 4475:

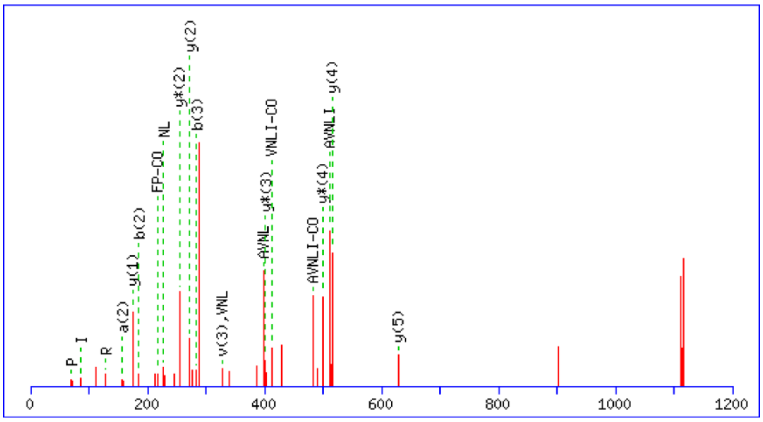

Spot 4478:

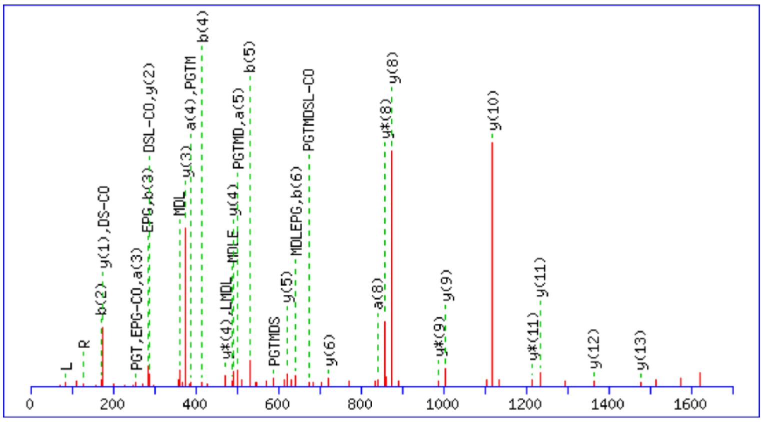

Spot 4230:

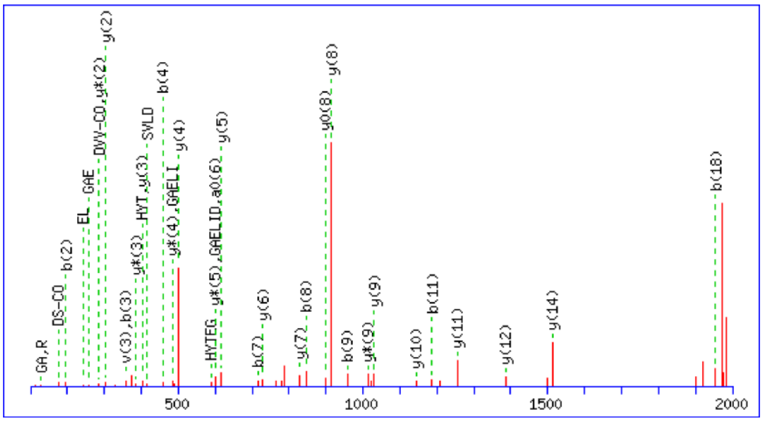

Spot 3551:

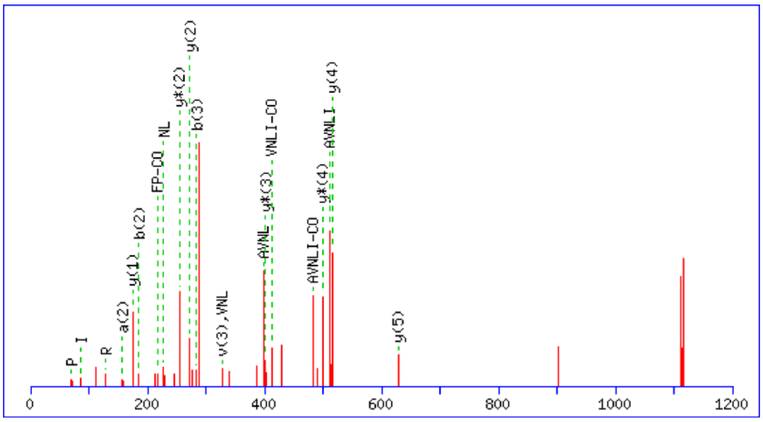

Spot 3554:

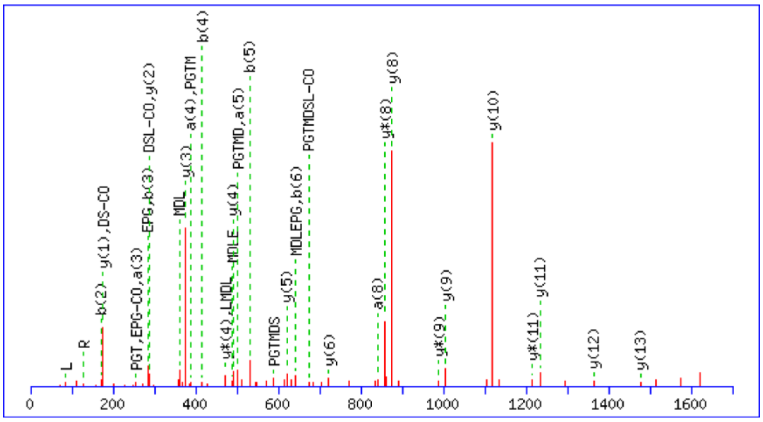

Spot 4460:

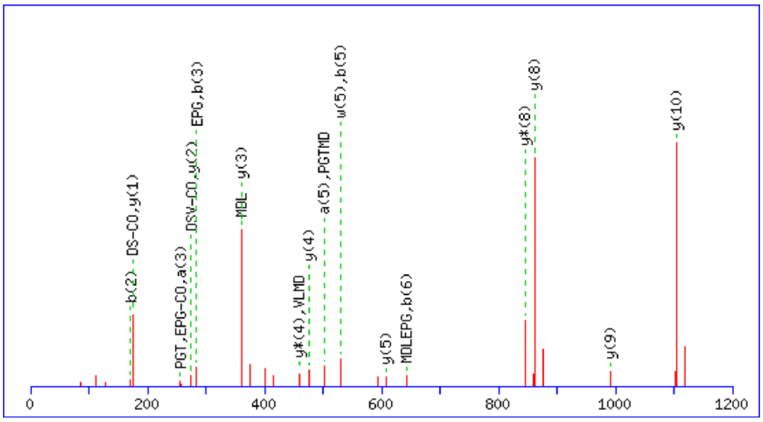

Spot 4462:

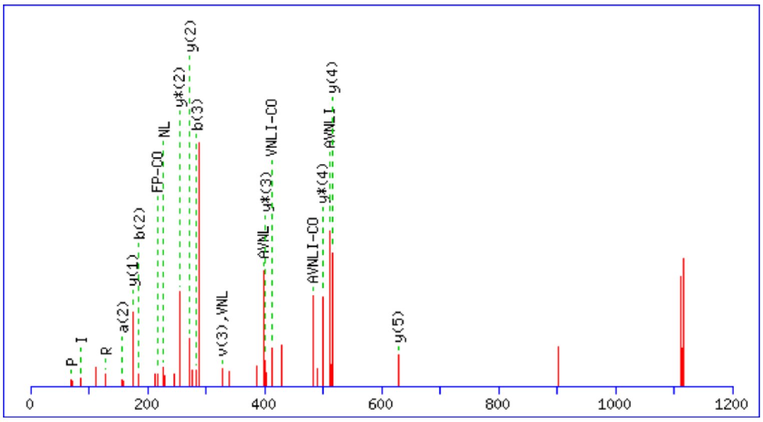

Spot 4465:

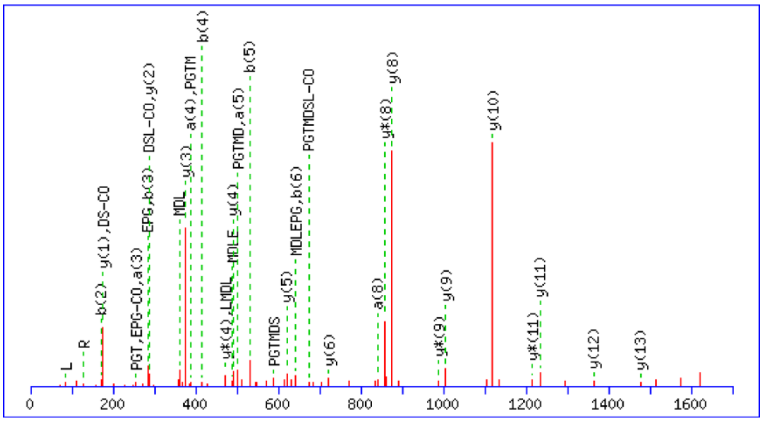

02-17-115:

A6:

Spot 679:

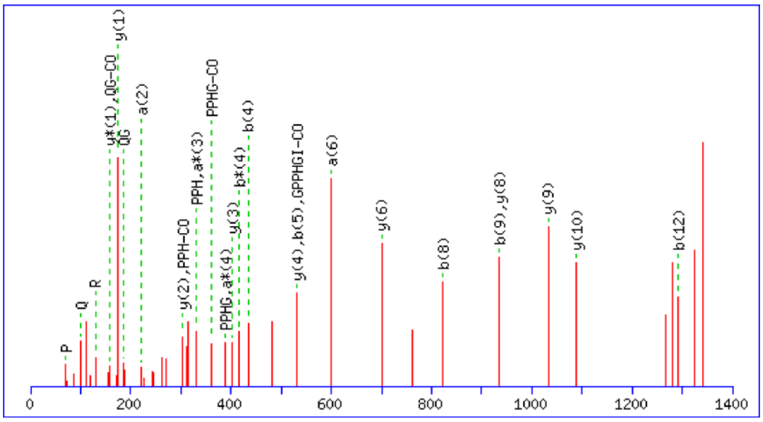

Spot 1085:

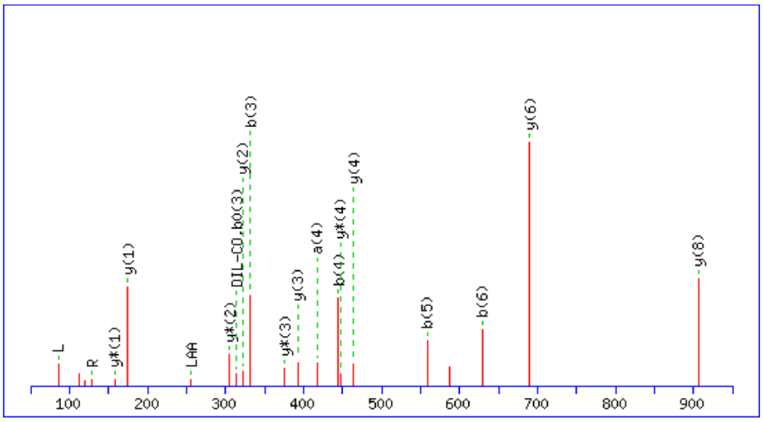

Spot 811:

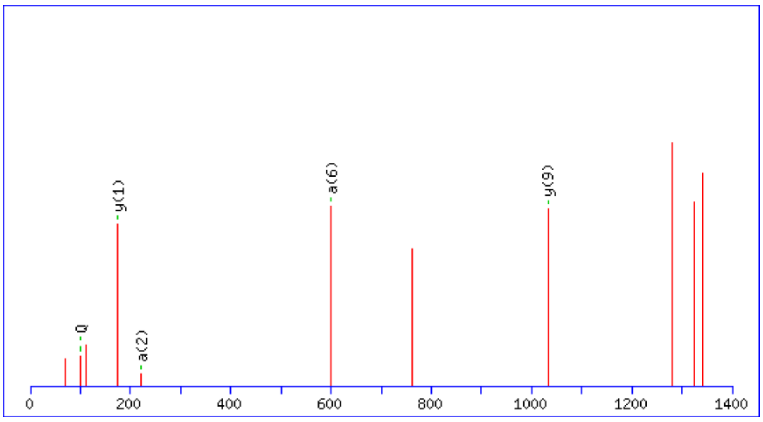

A33:

Spot 542:

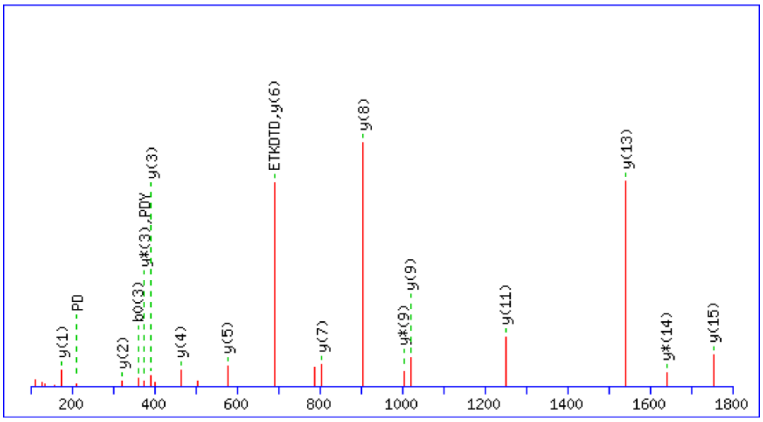

Spot 1088:

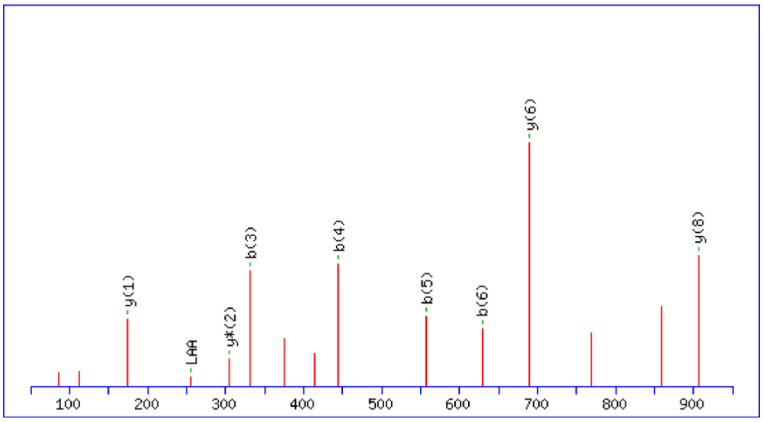

Spot 887:

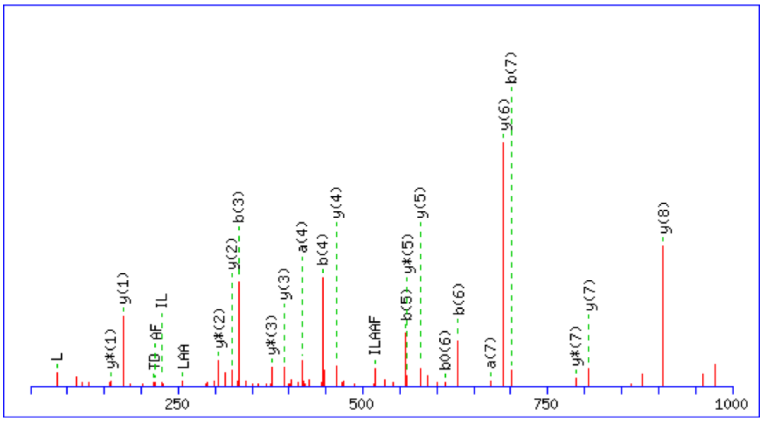

Spot 694:

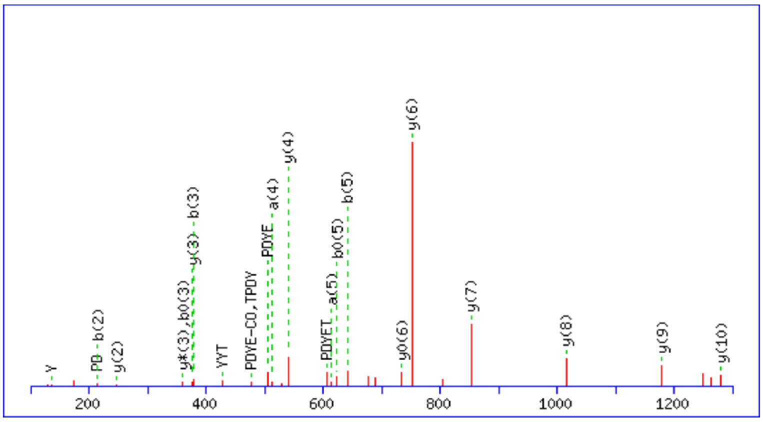

A48:

Spot 694:

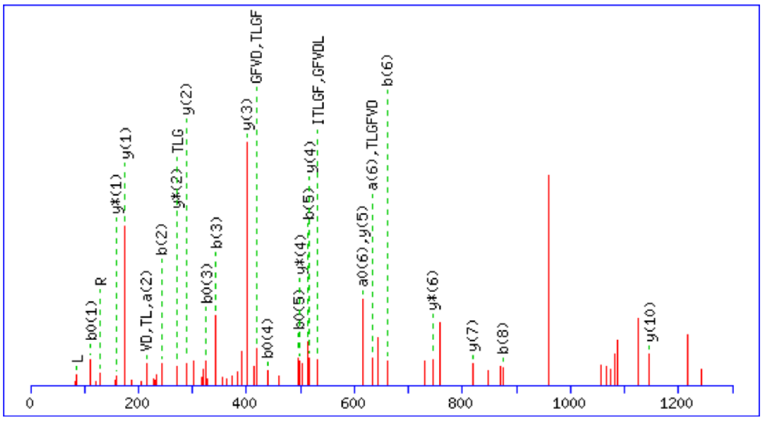

Spot 696:

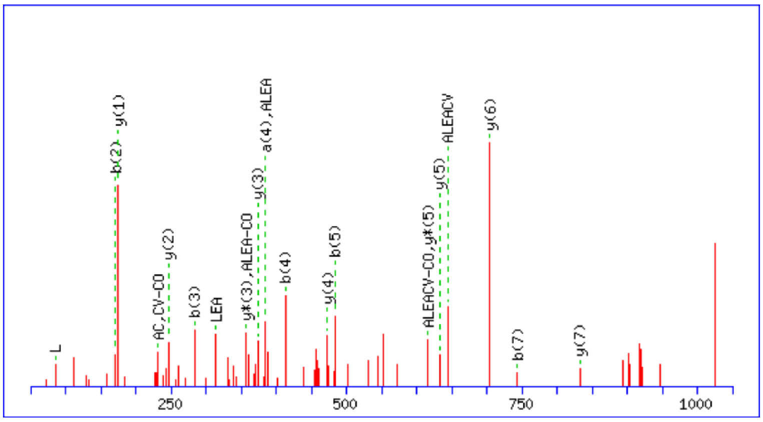

Spot 697:

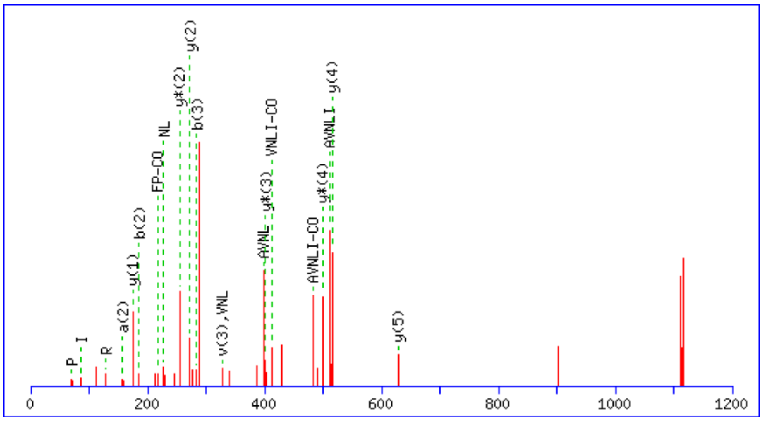

Spot 1091:

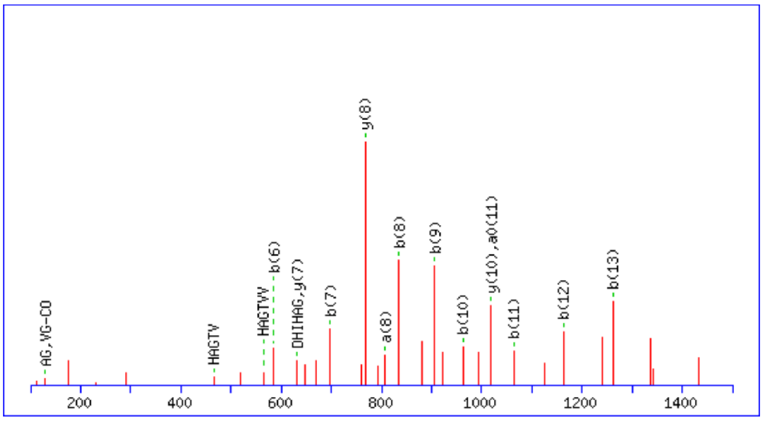

Spot 1095:

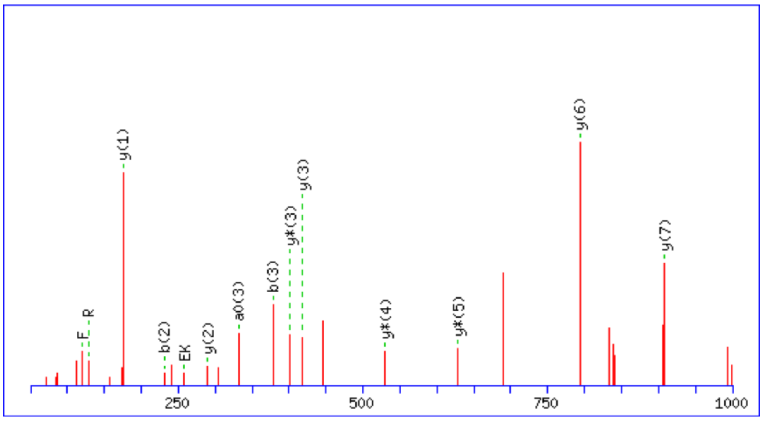

Spot 1097:

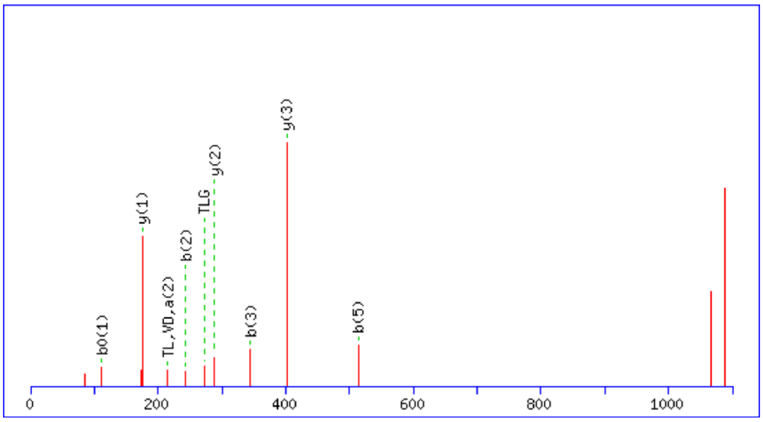

Spot 814:

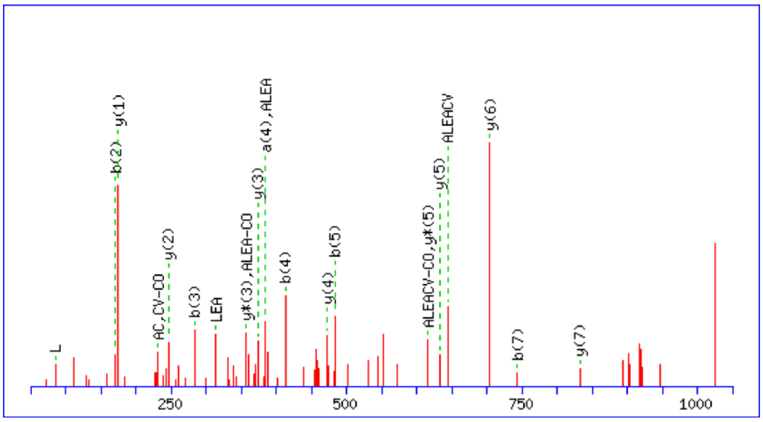

Spot 721:

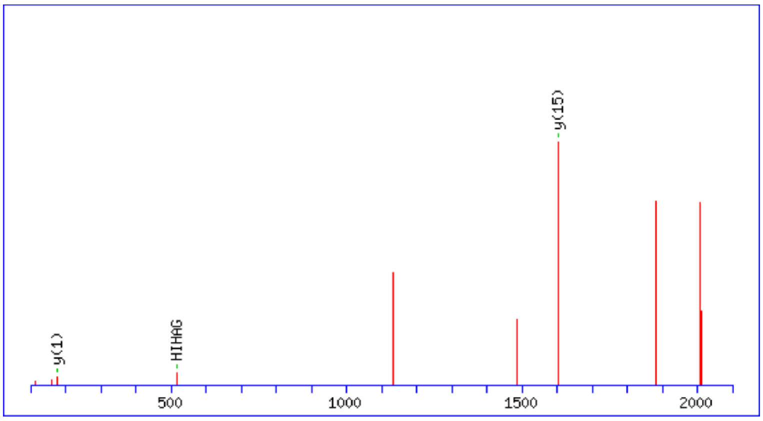

B38:

Spot 686:

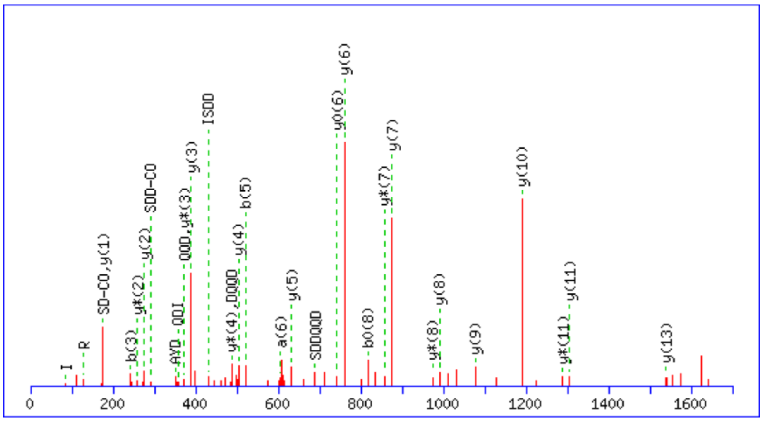

Spot 350:

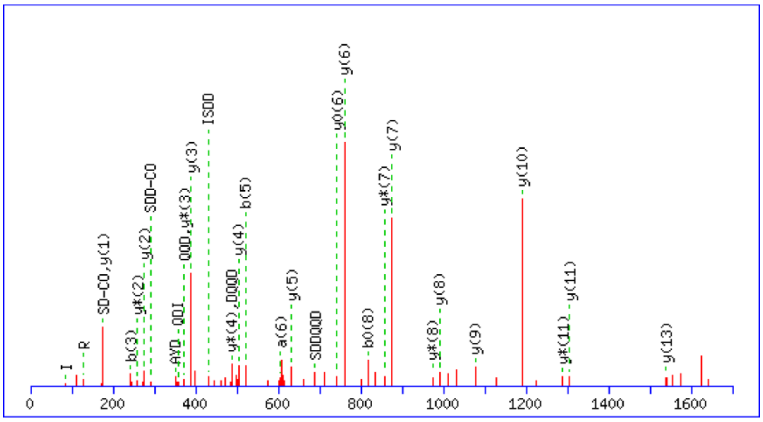

Spot 687:

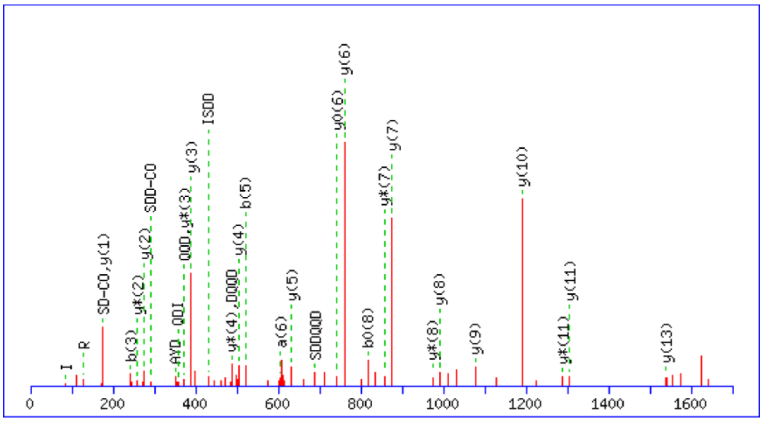

Spot 507:

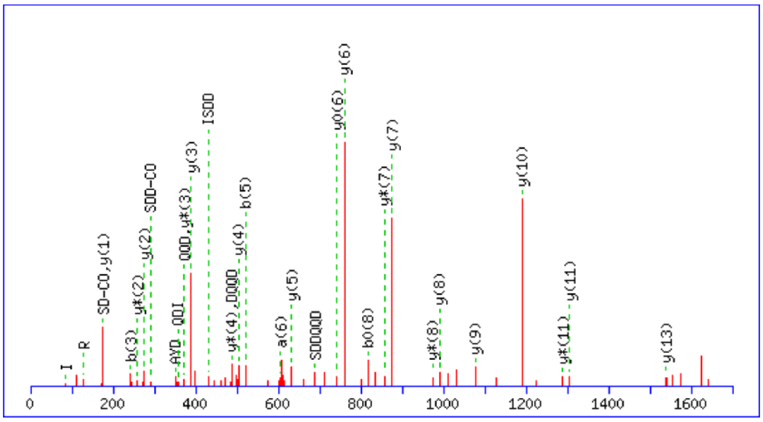

Spot 509:

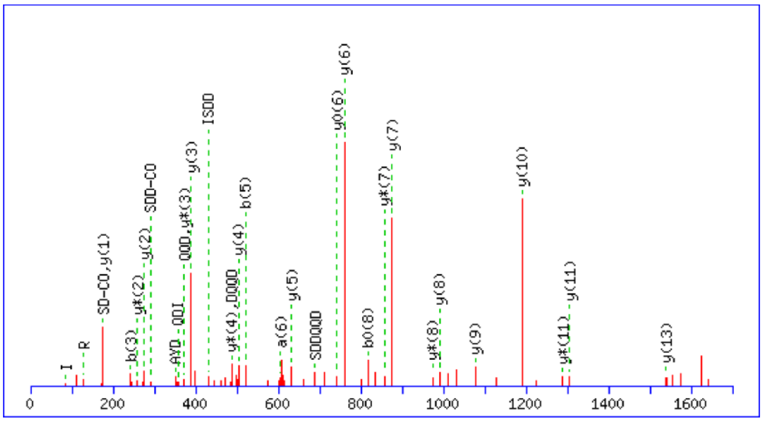

Spot 527:

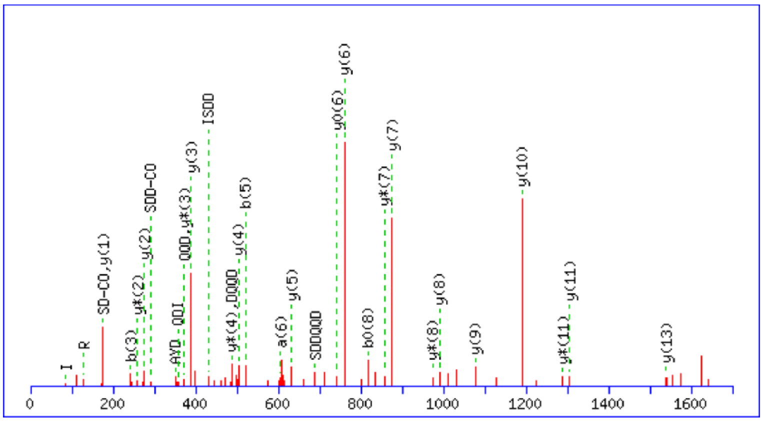

Spot 341:

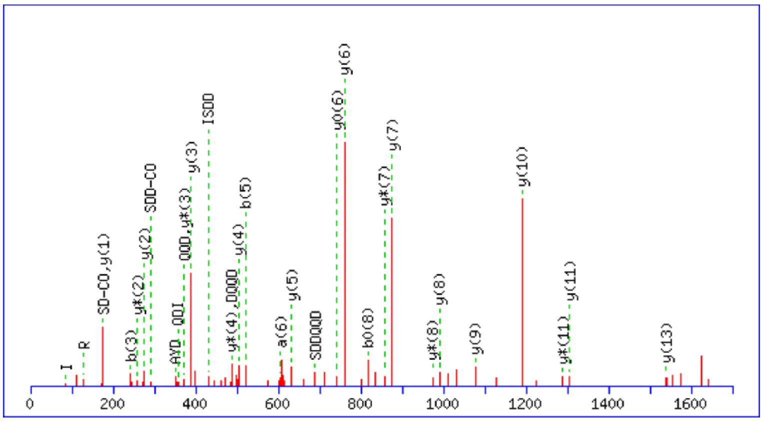

Spot 342:

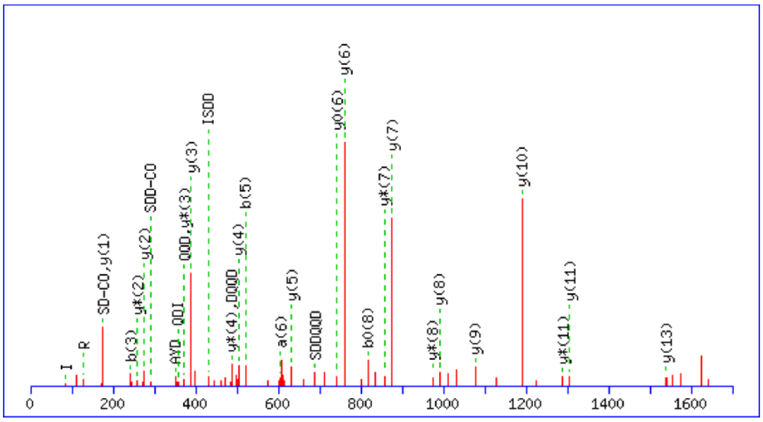

Spot 336:

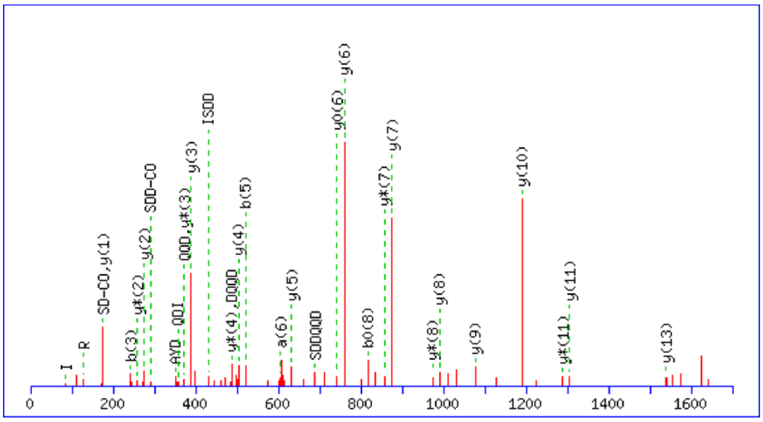

C24:

Spot 683:

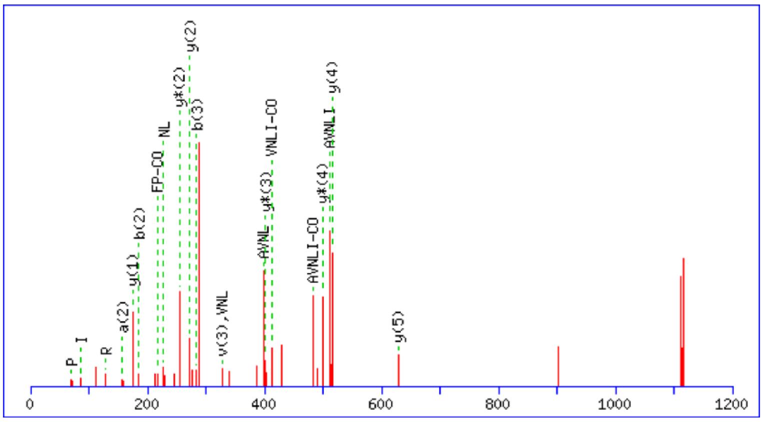

Spot 284:

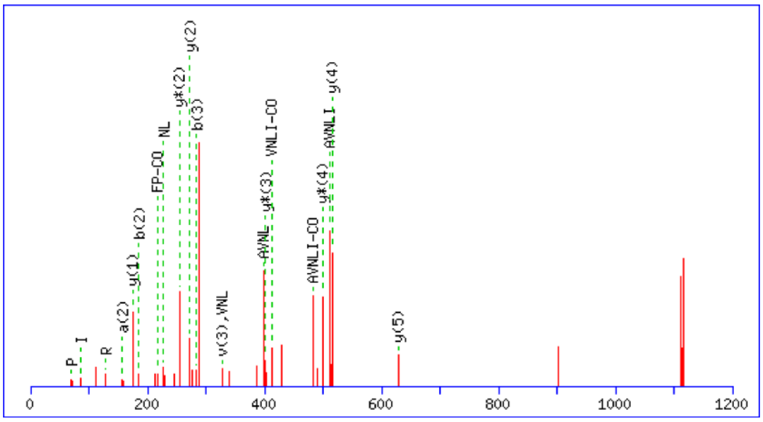

Spot 285:

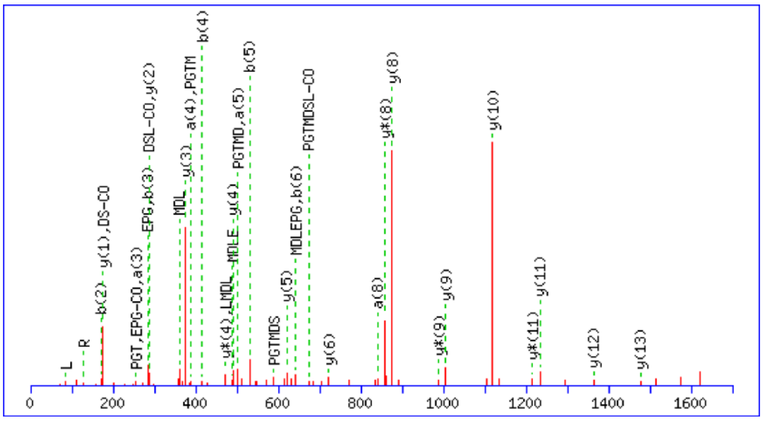

Spot 417:

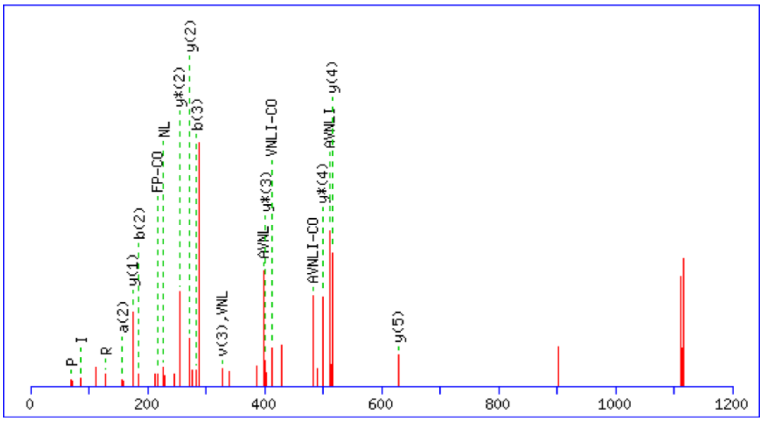

Spot 1084:

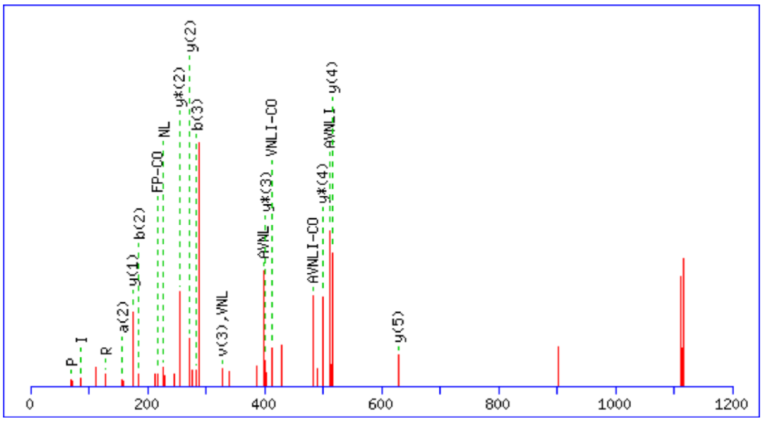

Spot 422:

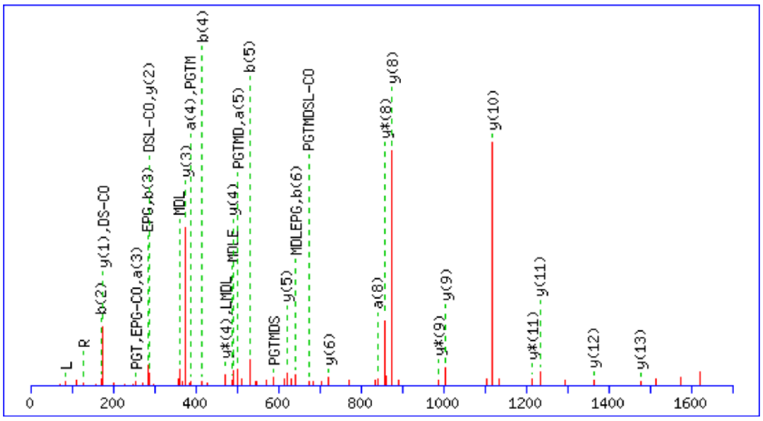

Spot 243:

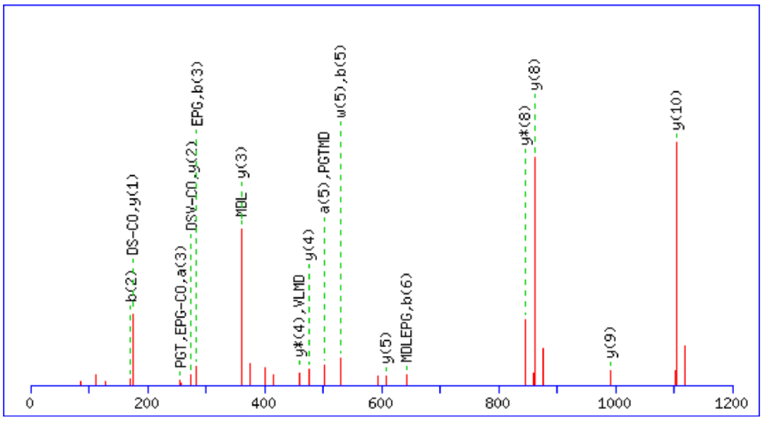

Spot 247:

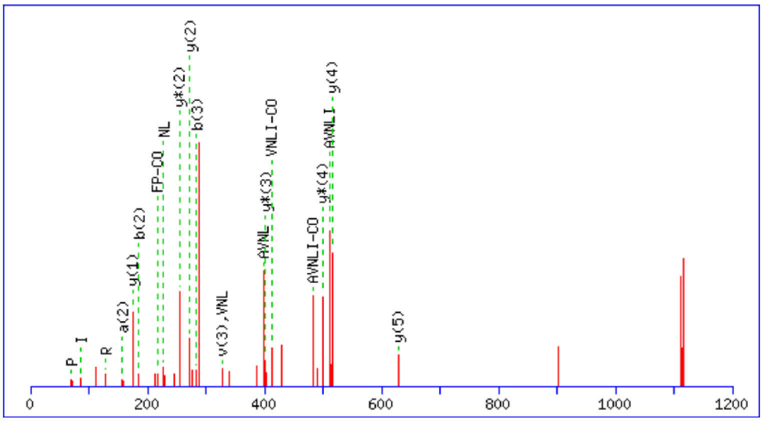

Spot 249:

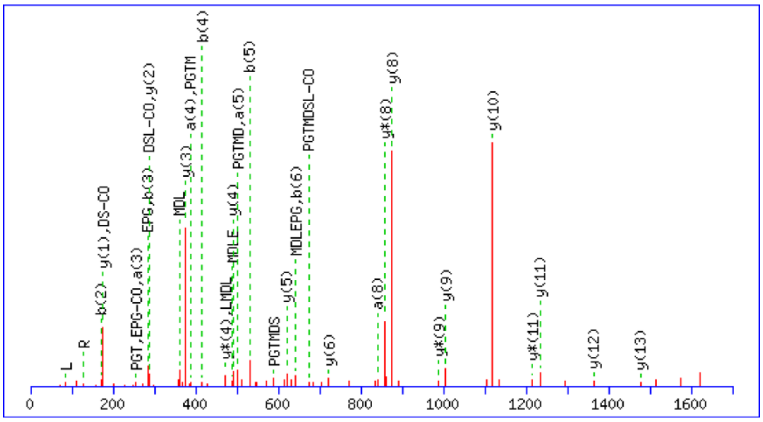

07-07-133:

A6:

Spot 420:

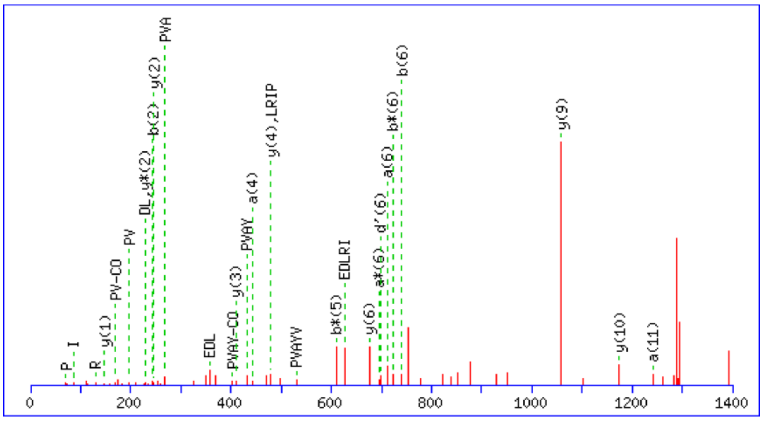

Spot 302:

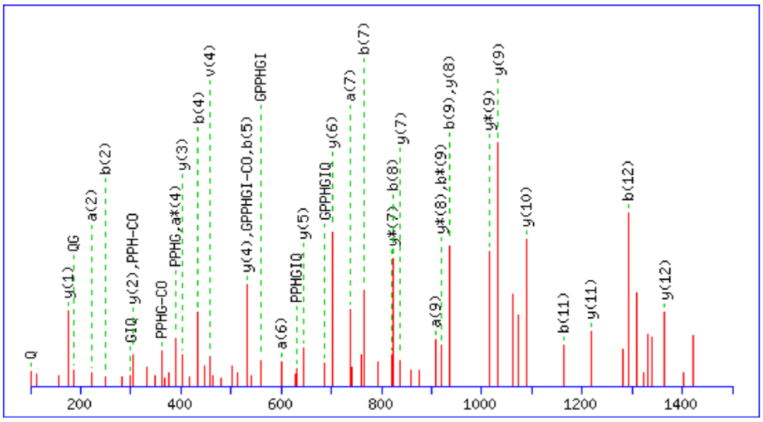

Spot 312:

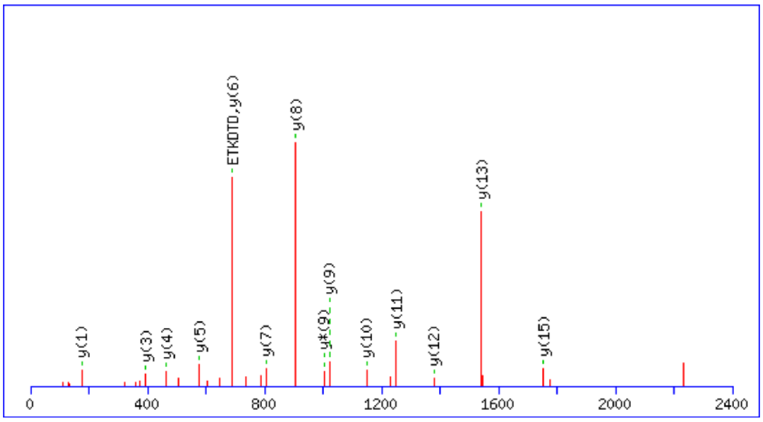

A33:

Spot 345:

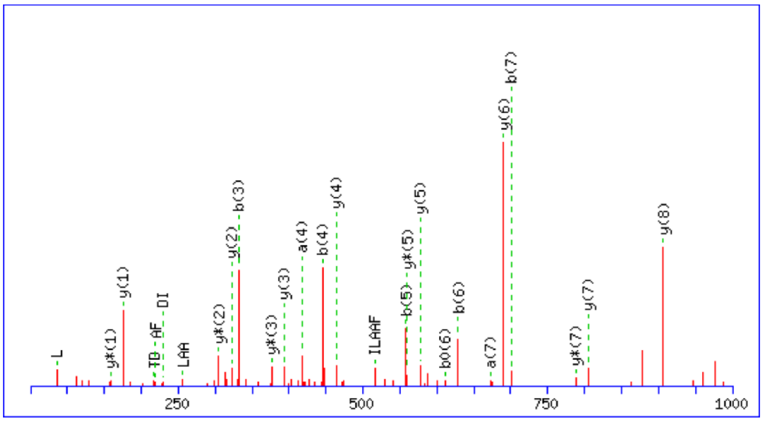

Spot 348:

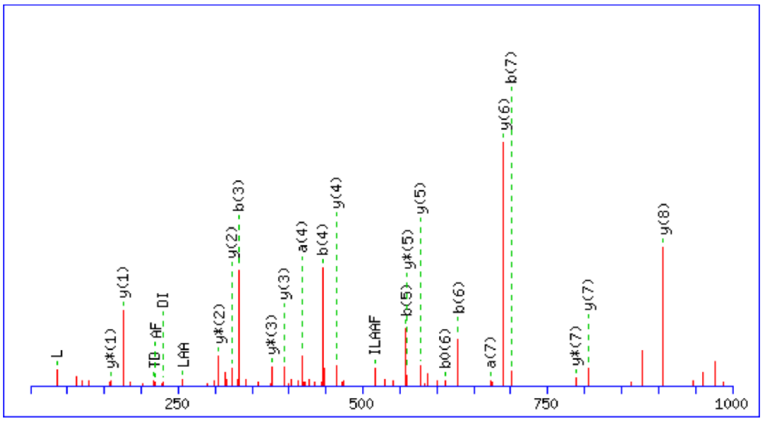

Spot 346:

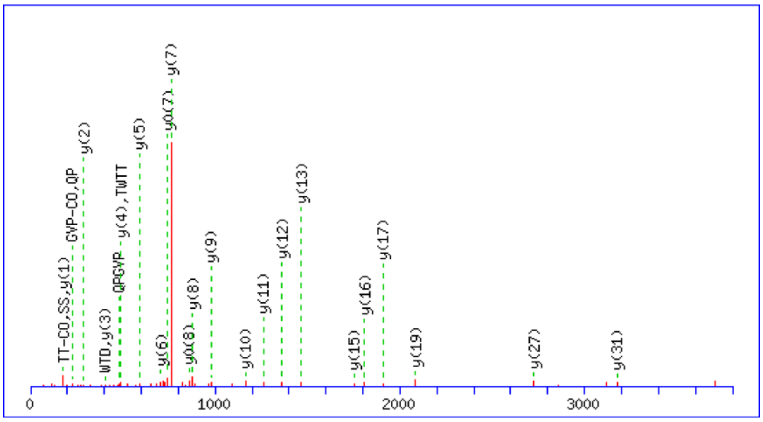

Spot 245:

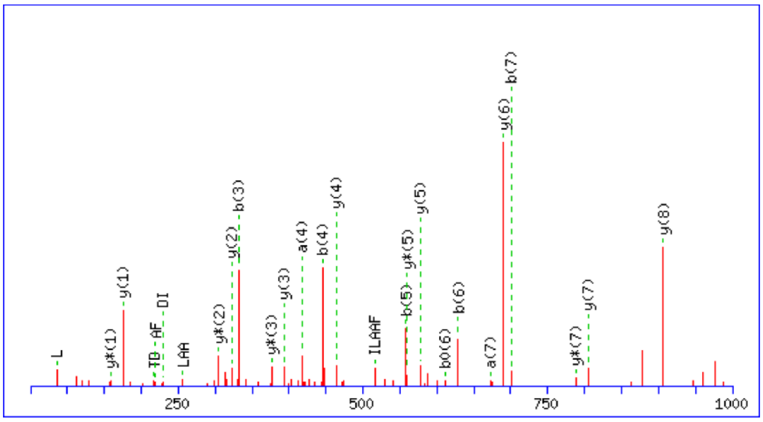

Spot 247:

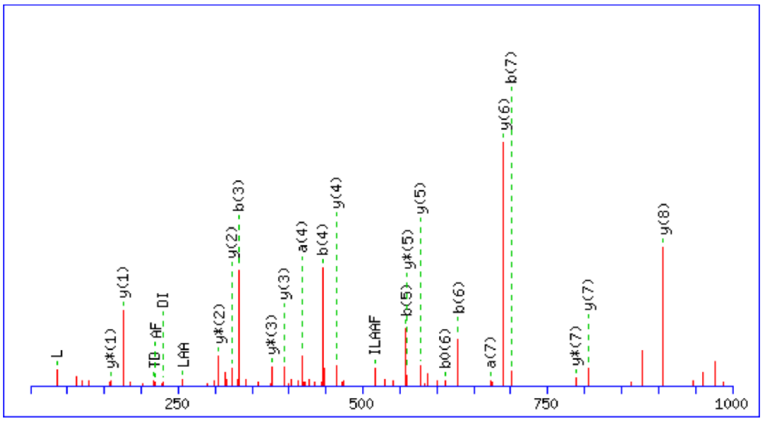

Spot 244:

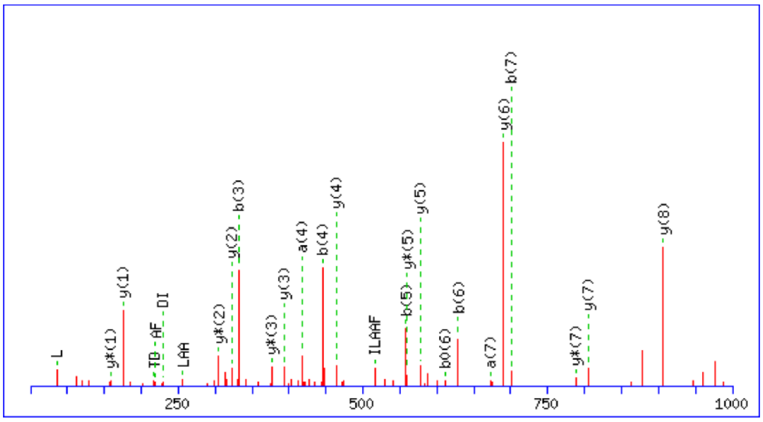

Spot 182:

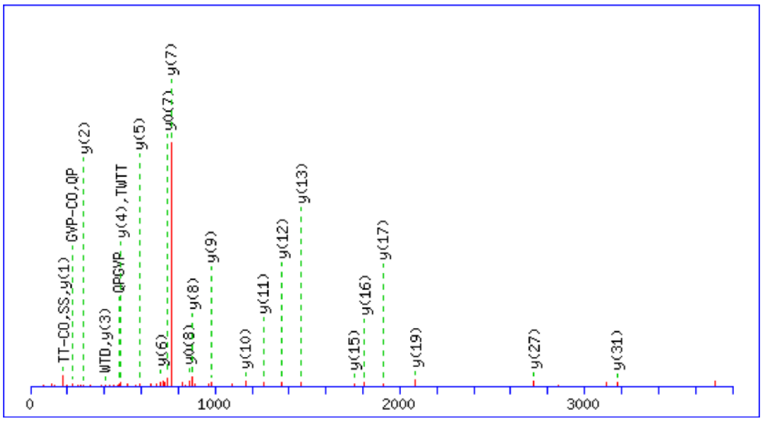

Spot 183:

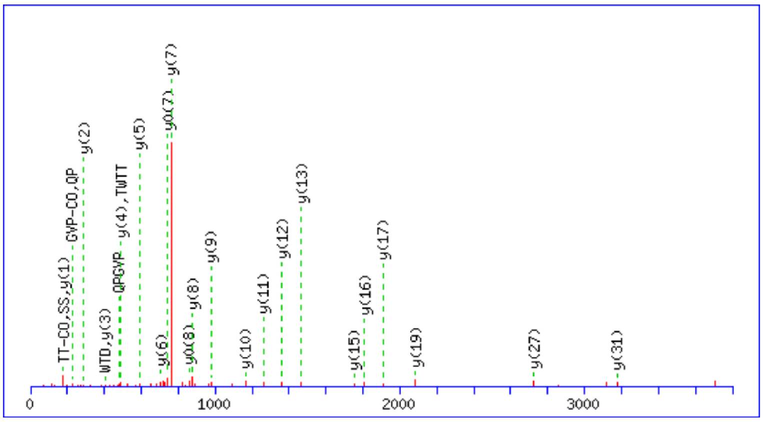

Spot 180:

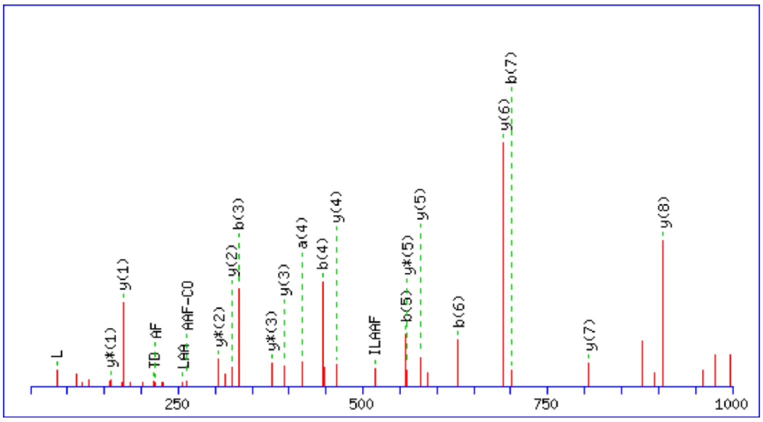

A48:

Spot 428:

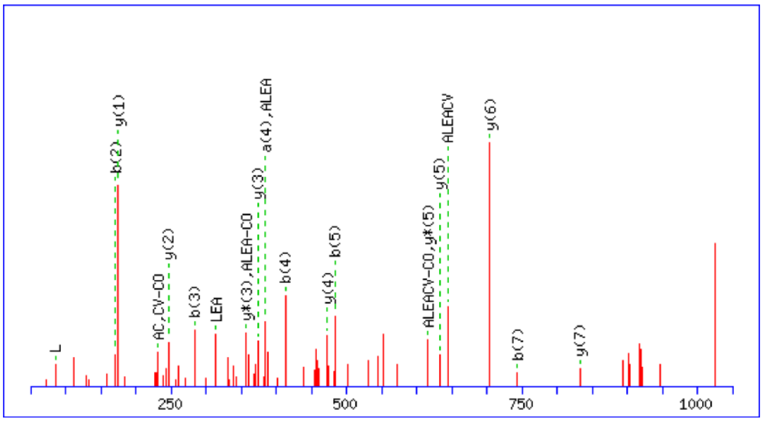

Spot 433:

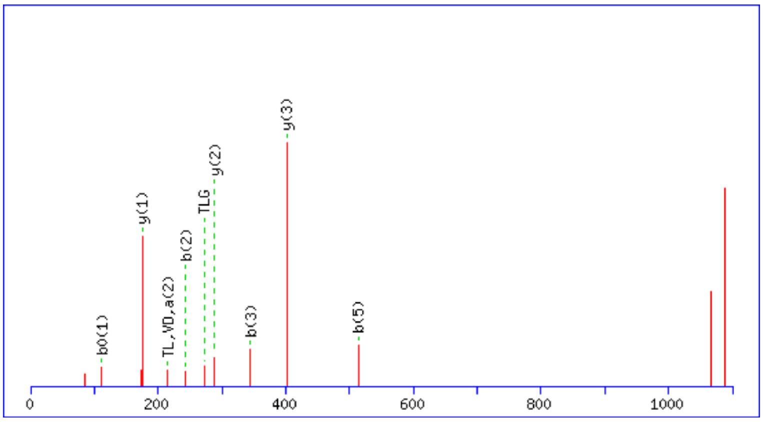

Spot 435:

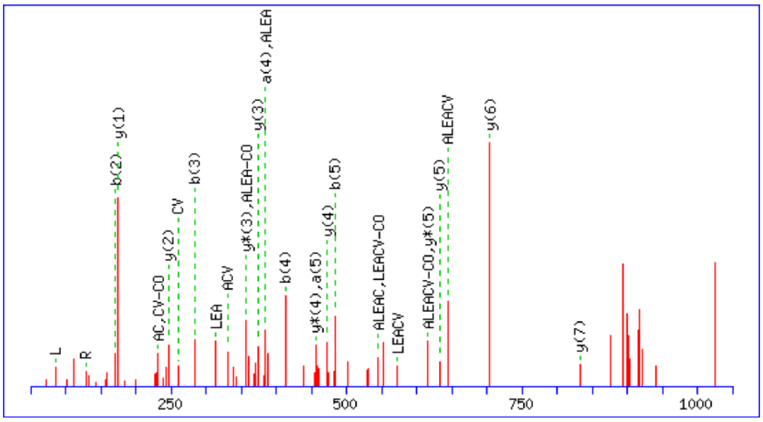

Spot 293:

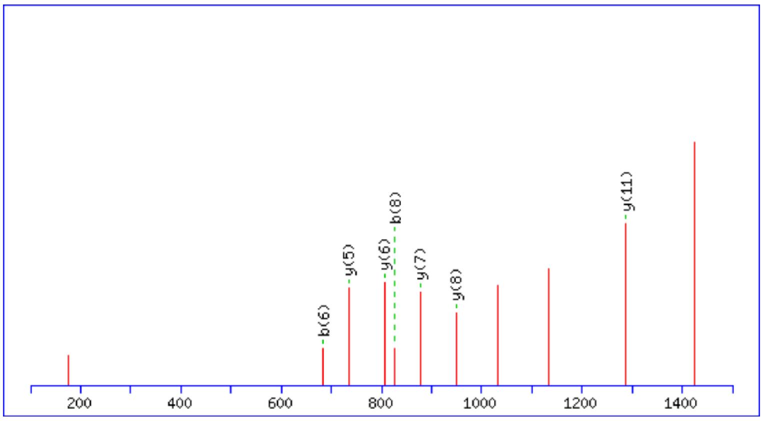

Spot 294:

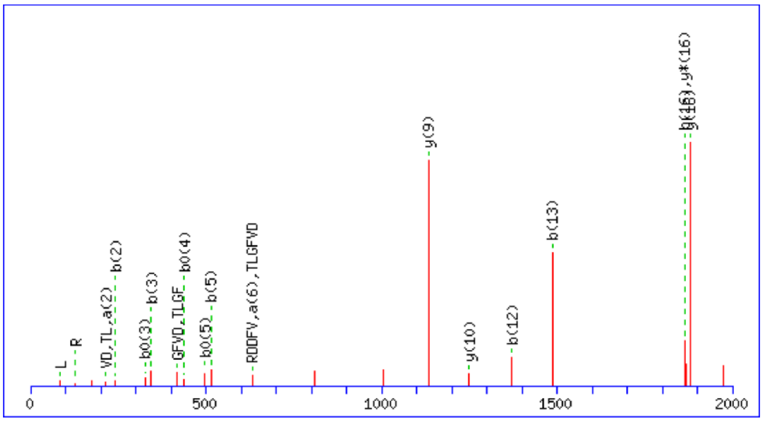

Spot 295:

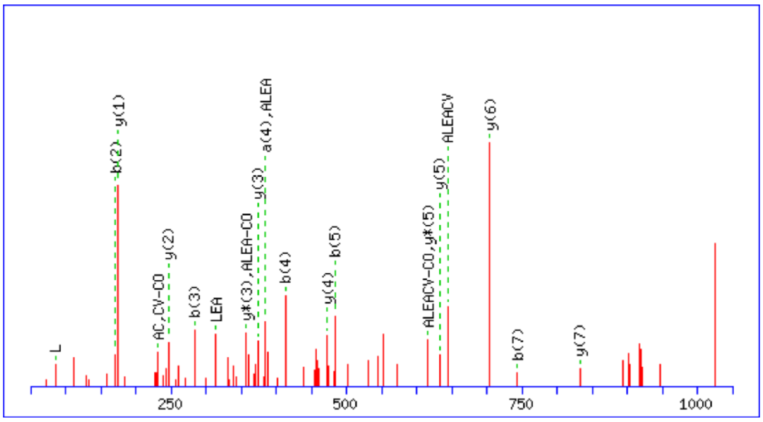

Spot 325:

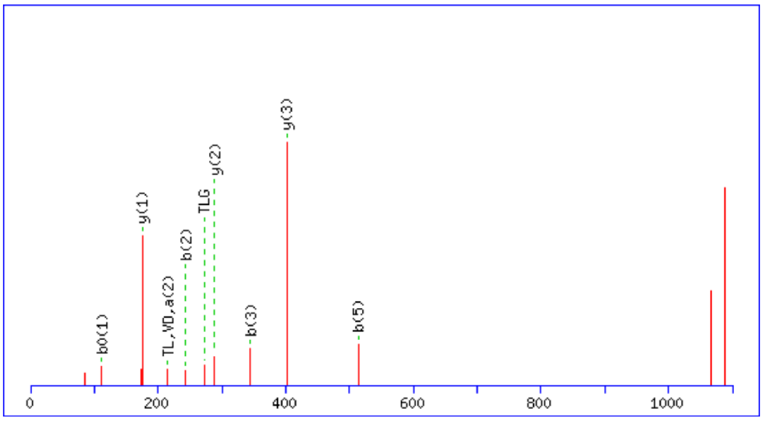

Spot 326:

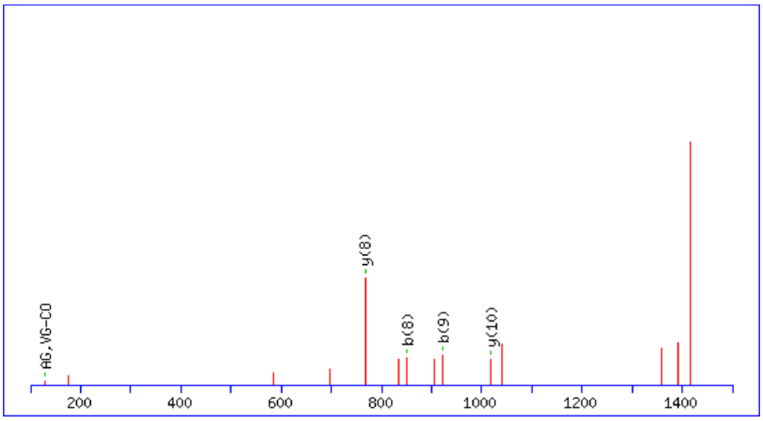

Spot 327:

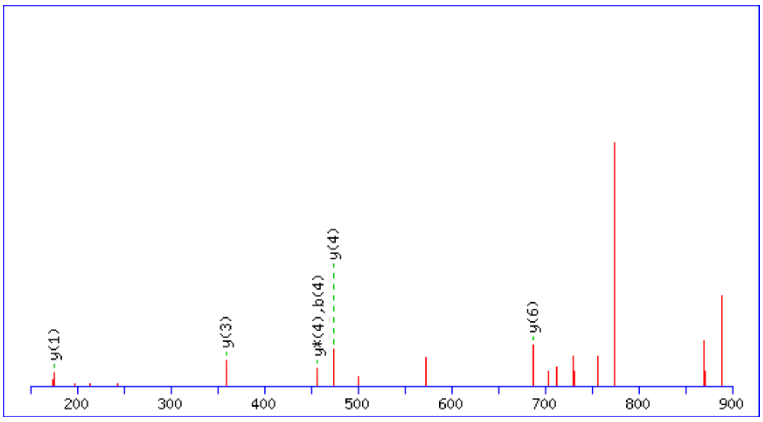

B38:

Spot 132:

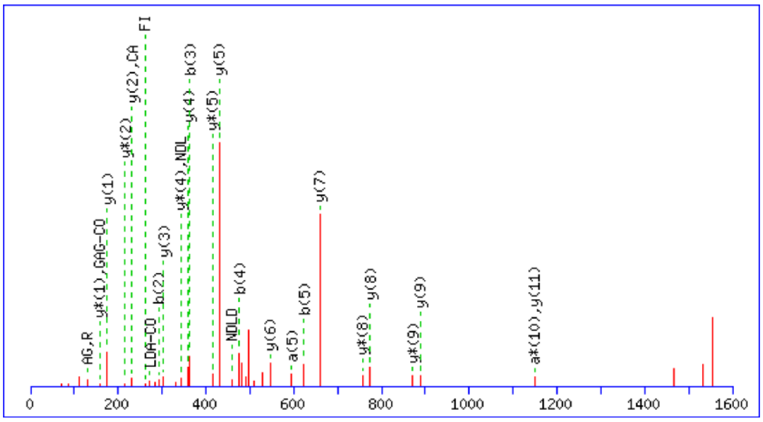

Spot 127:

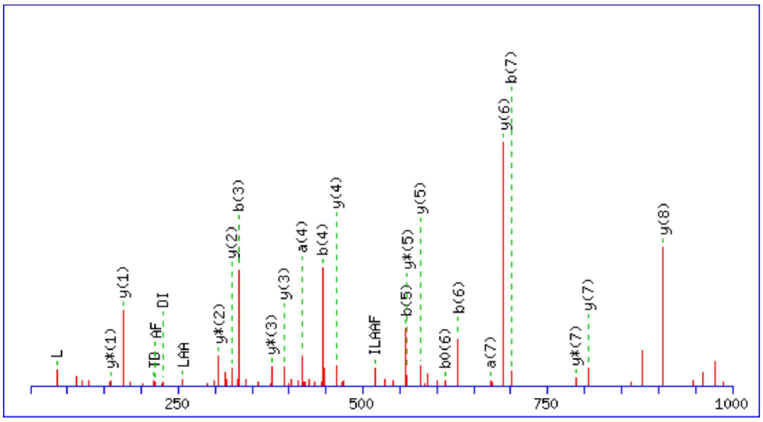

Spot 124：

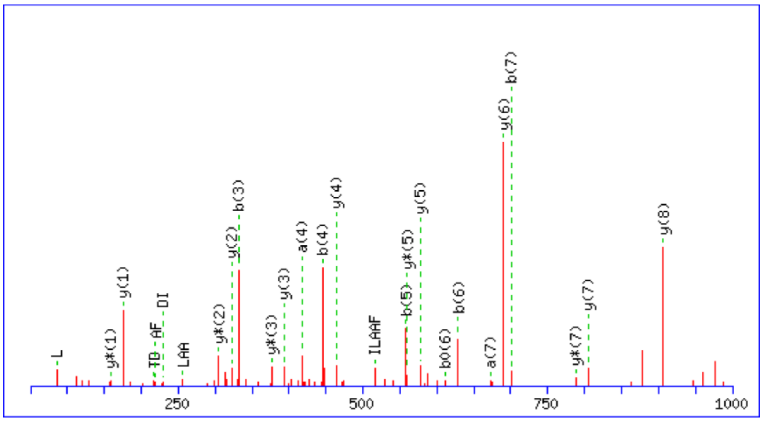

Spot 72：

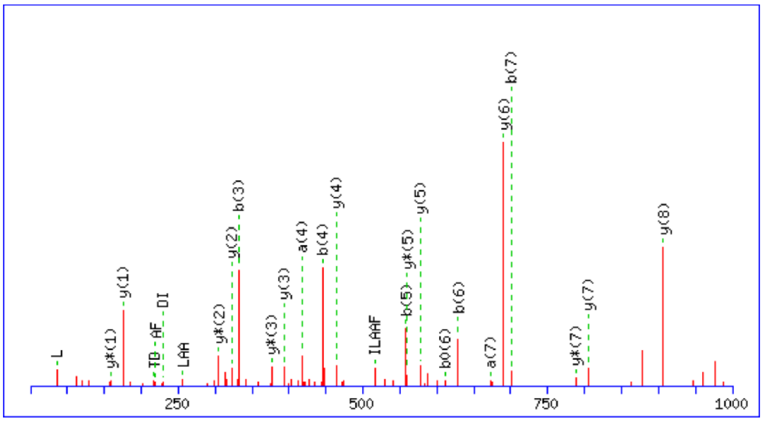

Spot 71：

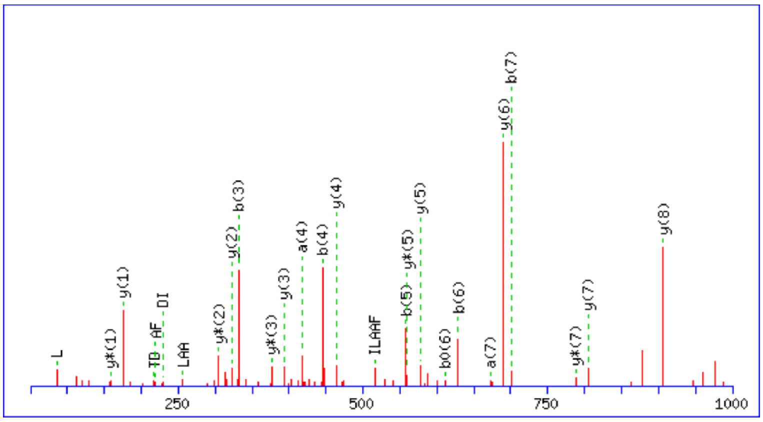

Spot 69：

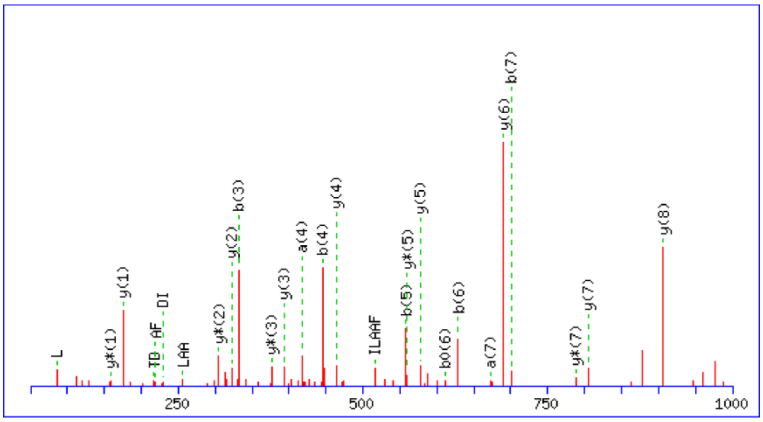

Spot 53：

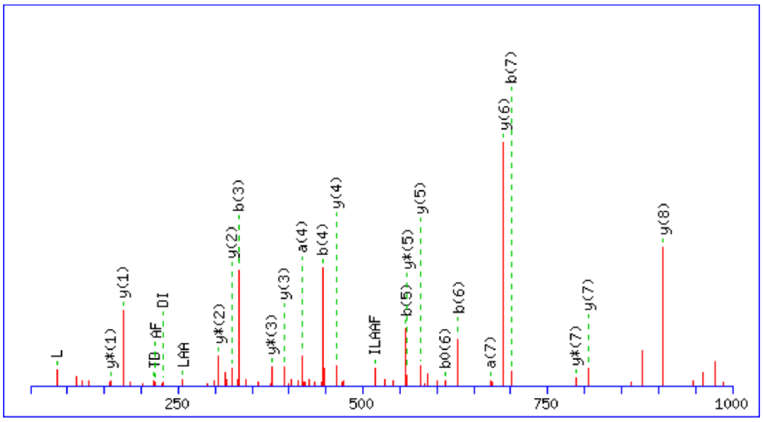

Spot 50：

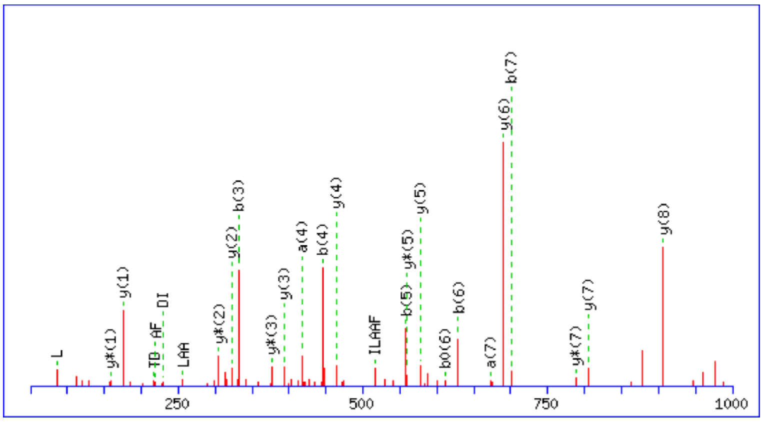

Spot 49：

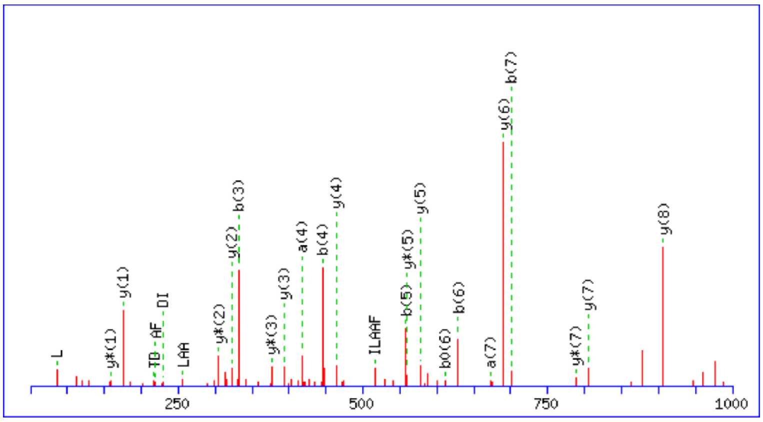

C24：

Spot 422：

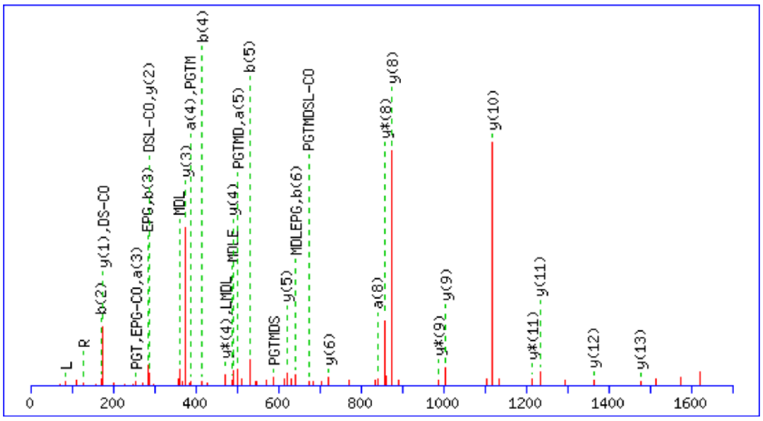

Spot 425:

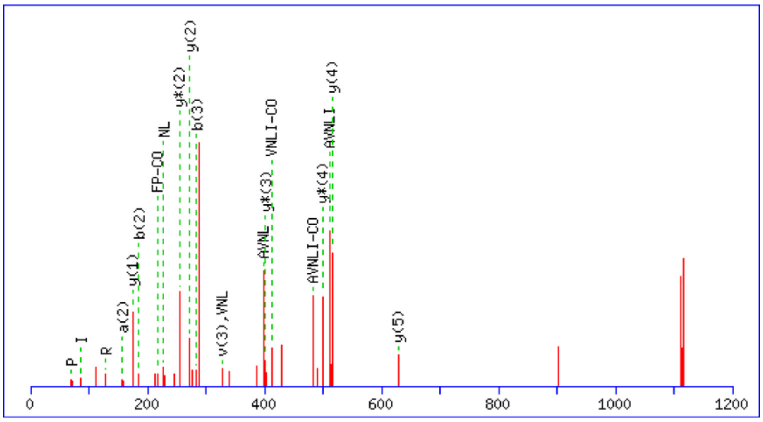

Spot 426:

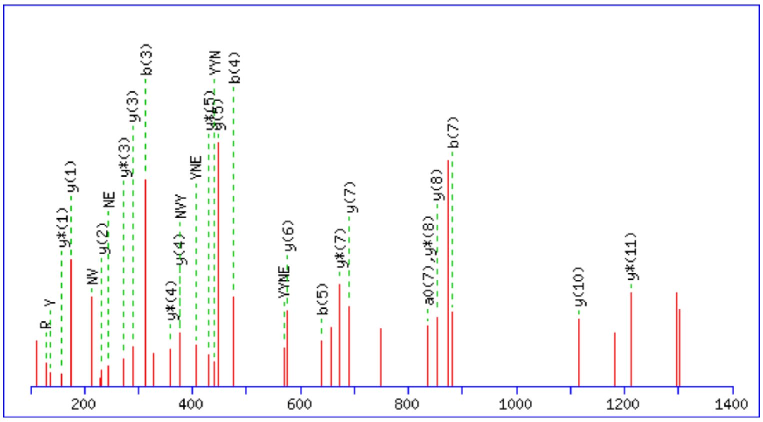

Spot 304:

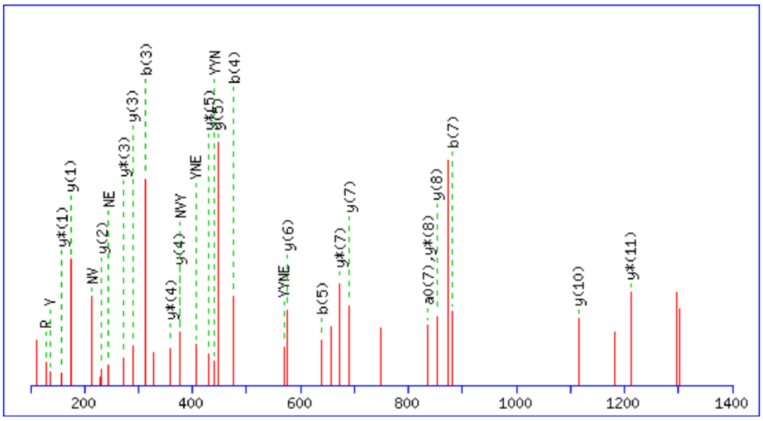

Spot 306:

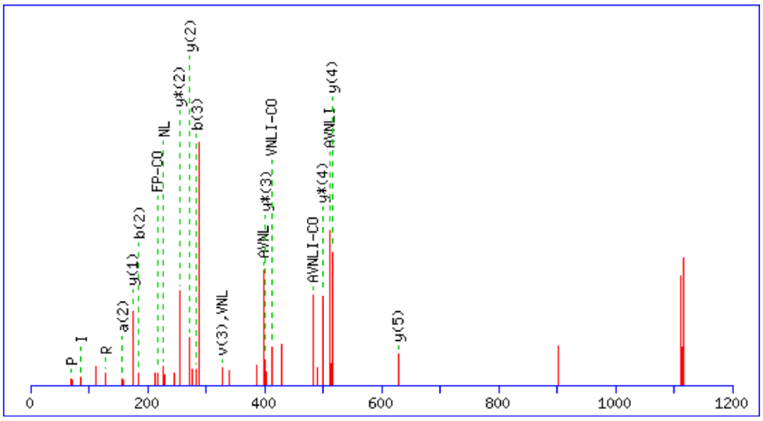

Spot 47：

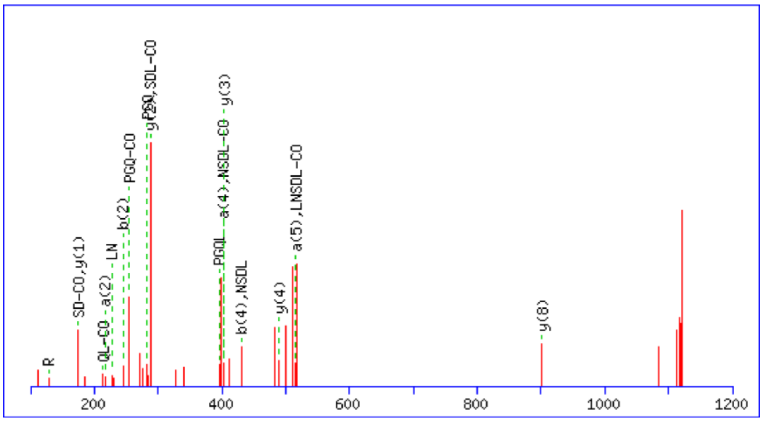

Spot 316：

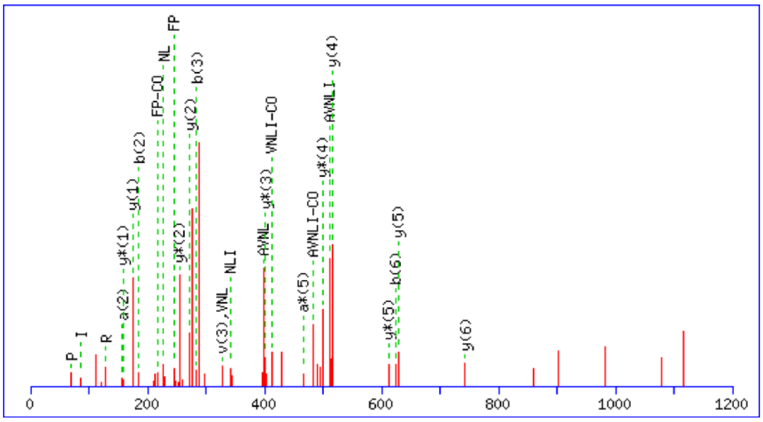

Images of 02-18-081, 02-17-115 and 07-07-133 are shown in Figures 1 and 2 and Figures S1, S2, S4, S5.
